# Curcumin derivative ST09 modulates the miR-199a-5p/DDR1 axis and regulates proliferation and migration in ovarian cancer cells

**DOI:** 10.1038/s41598-021-02454-1

**Published:** 2021-11-26

**Authors:** Febina Ravindran, Jinsha Koroth, Meghana Manjunath, Suchitra Narayan, Bibha Choudhary

**Affiliations:** 1 Institute of Bioinformatics and Applied Biotechnology, Electronic City Phase 1, Bangalore, Karnataka India; 2 Manipal Academy of Higher Education, Manipal, India

**Keywords:** Cancer genomics, Cancer therapy, Gynaecological cancer, Oncogenes, Computational biology and bioinformatics, Drug regulation, Target identification

## Abstract

Ovarian cancers are among the fatal malignancies affecting women globally, mainly due to their metastatic and chemoresistant nature. In this study, we report a potent curcumin derivative ST09 effective against ovarian cancers. Prior in-vitro studies with ST09 drug showed cytotoxicity in tumorigenic cells compared to normal cells and in-vivo, significant tumor reduction was observed with least systemic toxicity. ST09 induced cytotoxicity in the ovarian cancer cells triggering mitochondria-mediated intrinsic apoptotic pathway. Delving deeper to understand the underlying molecular mechanisms involved in ovarian cancer pathogenesis, we identified an inverse correlation of miR-199a-5p with DDR1, a collagen receptor with receptor tyrosine kinase activity. The ST09 treatment in ovarian cancer cell lines resulted in the deregulation of the miR-199a-5p/DDR1 axis, conferring tumor-suppressive functions. We established DDR1 to be a direct target of miR-199a-5p and that ST09-induced DDR1 loss in these ovarian cancer cells resulted in the inactivation of its downstream MMP activation, migration, EMT, and prosurvival NF-κB pathway. Overall this study demonstrates ST09, a potent drug candidate for ovarian cancer treatment which exhibits anti-invasive and migrastatic properties.

## Introduction

The global cancer burden is spiraling, and the pursuit for a potent anti-cancer drug with minimal adverse effects has been challenging for drug discoverers. Current chemotherapeutic drugs used for cancer treatment are effective against cancerous cells but toxic to normal cells resulting in harmful and long-term side effects like nervous disorders, cardiotoxicity, neutropenia, and nephrotoxicity^1^. Many plant-derived natural compounds have shown chemopreventive and anti-carcinogenic effects for several cancer types alleviating toxic side effects^2^.

Curcumin (diferuloylmethane) is a natural compound derived from the spice plant *Curcuma longa* L.*,* exhibiting anti-cancer properties against broad cancer types^3^. It is also known for its anti-microbial, anti-inflammatory, anti-proliferative, anti-angiogenic, antimutagenic, and antioxidant properties^4^. It is reported to exert its anticancer activity via acting on transcription factors/signaling cascades involved in cellular proliferation, apoptosis-inducing factors, autophagy inducing factors, adhesion molecules, and modulating oncogenic/tumor-suppressive miRNAs^5^. Curcumin entered phase I and phase II clinical trials for anti-cancer therapies as monotherapy and combinational therapy. However, despite its safety at high doses and great tolerance, its poor solubility, poor systemic bioavailability, and rapid metabolism, unfortunately, hindered its clinical utility^6^.

Aimed at enhancing curcumin’s therapeutic efficacy, we developed a series of novel curcumin derivatives denoted ST03, ST06, and ST08 derived from dimers of diarylidenyl-piperidones (DAP). Dimers of DAP are synthetic curcumin analogs reported to have anti-cancer properties with superior stability and bioavailability compared to curcumin^7^. We have reported the cytotoxic effect of ST06 in-vitro on cervical cancer, and in-vivo studies showed significant tumor reduction in mouse models^8^. The cytotoxic effects of ST03 and ST08 were tested in-vitro on breast and ovarian cancer cell lines. However, the mechanism for cellular cytotoxicity was not investigated^9^. Another novel curcumin derivative, ST09 (1,2-bis((3E,5E)-3,5-bis(4-chlorobenzylidene) -4-oxopiperidine-1-yl)ethane-1,2-dione hemihydrate) was synthesized by the addition of 4-chlorobenzylidene moiety to the DAP dimer backbone and is a structural isomer of ST03. The cytotoxic effects of ST09 were studied in breast cancer cells. It resulted in apoptotic cell death by activating intrinsic apoptotic pathway, and in-vivo studies showed a significant reduction in tumor growth in mice (EAC model) with no adverse systemic toxicity^10^.

The role of the extracellular matrix in driving cancer progression and metastasis is very well documented^11^. One of the most abundant proteins in the ECM is collagen. Collagen signals via its receptors Discoidin domain receptors (DDR1 and DDR2) which belong to the subfamily of tyrosine kinases. Also, DDR1 signaling in tumors aids in tumor progression, metastasis, and resistance to chemotherapy^12^. Therefore, DDRs have been attractive targets for therapy. In this regard, DDR inhibitors have been developed to inhibit collagen-mediated chemoresistance observed in resistant tumours^13^. Collagen IV mediated DDR1 activation led to increase in MMP-9 and collagen IV establishing a feed-forward loop which promoted the migration and adhesion of myeloid leukemia cells in bone marrow by activating AKT pathway^14^. In ovarian cancer cells, the binding of COL11A1 to DDR2 activated PI3K/AKT-NFκB signaling pathways which induced apoptosis in ovarian cancers^15^. In cancers such as hepatocellular carcinoma and acute myeloid leukemia, DDR1 expression has been shown to be anti-correlated with miR-199a^16^. It is becoming evident that in cancer cells, the aberrant expression of genes is related to deregulated miRNA.

miRNAs are a class of small, noncoding RNAs that regulate gene expression by targeting mRNA by binding at their 3’UTR. There are two fates of the miRNA bound mRNA; either mRNA degrades (transcriptional block) or a translational block can occur both leading to the absence of the protein^17^. miRNA deregulation affects a variety of physiological and pathological processes in cancer. In this context, the role of miRNAs in tumor growth, angiogenesis, and metastasis^18^, as well as their use as diagnostic and therapeutic biomarkers is well established^19^. A single miRNA can bind to the UTRs of several genes and regulate their expression and the global impact of miRNA on mRNA has been studied by integrating miRNA and mRNA expression levels. Drug induced changes at the cellular level are mostly carried out using transcriptome sequencing^20^. Understanding the drug induced changes by integrating miRNA and mRNA is essential for devising future therapeutics based on the drugs' chemosensitivity/chemoresistance and toxicity.

This study aimed to investigate the sensitivity of ST09 against ovarian cancers and its global impact on miRNA and mRNA expression using next-generation sequencing technology. To address the molecular and cellular changes induced by ST09 against ovarian cancers, we used two different ovarian cancer cell lines for this study; PA1, an ovarian teratocarcinoma derived cell line which is undifferentiated with stem cell like characteristics, and A2780 an endometrioid ovarian adenocarcinoma derived cell line which is differentiated with epithelial characteristics.

## Materials and methods

### Cell culture

Ovarian cancer cell lines PA1 and A2780 were purchased from NCCS (Pune, India) and ATCC (USA). PA1 cells and A2780 cells were cultured in EMEM (Lonza) and RPMI-1640 (Lonza) media, respectively, supplemented with 10% FBS and maintained as monolayer cultures at 37 °C with 5% CO_2_ in a humidified incubator. The drug ST09 was reconstituted in DMSO and used at different concentrations for various studies. Vehicle control is denoted as the control in this study, where DMSO was used for treatment at 0.02% (v/v).

### MTT assay

Both PA1 and A2780 cells were plated at a density of 5000 cells/well in a 96-well plate overnight, treated with increasing concentrations of ST09 drug, and further incubated for 24 or 48 h. Post incubation, cells were treated with MTT (MP Biomedicals) at a concentration of 0.5 mg/ml and incubated till the purple colour of formazan was visible. The formazan crystals were solubilised by adding 50% DMSO solution and incubated for 2 h at 37 °C. Post solubilisation, the absorbance was measured at 590 nm and bar graphs plotted as percentage cell viability compared to untreated control.

### Apoptosis assay

Apoptosis induced by ST09 drug was evaluated using Annexin V-FITC/PI kit (Invitrogen). The assay was performed according to the manufacturer's protocol. Briefly, both PA1 and A2780 cells were treated with increasing concentrations of ST09 drug for 48 h, post incubation stained with Annexin V-FITC and PI, and analysed by flow cytometry (Gallios, Beckman Coulter). A minimum of 10,000 events were acquired per condition, data was analysed using Gallios software. Flow cytometry dot plots were generated using Gallios software (version 1.2, https://www.mybeckman.in/flow-cytometry/software/kaluza-for-gallios). 

### JC-1 assay

Mitochondrial staining kit (Sigma CS0390) was used to evaluate the mitochondrial membrane potential of ST09 drug-treated ovarian cancer cell lines. Briefly, 48 h ST09 treated PA1 and A2780 cells were harvested and stained with JC-1 (5,5′,6,6′-tetrachloro-1,1′,3,3′-tetraethyl-imida carbocyanine iodide) for 20 min at 37 °C. Post incubation, the JC1 fluorescence was measured by flow cytometry (Gallios, Beckman Coulter). 10,000 events were acquired, and the dot plot plotted against the green/red population fluorescence intensity using Gallios software. Flow cytometry dot plots were generated using Gallios software (version 1.2, https://www.mybeckman.in/flow-cytometry/software/kaluza-for-gallios).

### Plasmid construct, transfection, and luciferase assay

The 3’UTR of DDR1 was cloned into the pmirGlo vector downstream of the firefly luciferase gene and denoted pmirGlo-DDR1-3’UTR. For transfection, PA1 and A2780 were seeded at 20,000 cells/well in a 96-well plate and treated with ST09 drug for 16 h. The following day, both control and drug pre-treated cells were transfected with 1 µg pmirGlo-DDR1-3’UTR in Opti-MEM I Reduced Serum Medium (Thermo Fisher Scientific) using Lipofectamine 2000 (Thermo Fisher Scientific). The firefly luciferase activity was measured post 48 h of transfection using the luciferase assay system (Promega) and bar graph plotted against the firefly luminescence.

### Anti-mir transfection

The anti-miR-199a-5p was purchased from Ambion. The delivery of anti-miR-199a-5p in PA1 cells was performed using oligofectamine (Thermo Fisher Scientific). Briefly, PA1 cells at 70% confluency in 96-well or 6-well plates were transfected with anti-miR-199a-5p (2.5 nM) in Opti-MEM I Reduced Serum Medium (Thermo Fisher Scientific). The following day, the cells were treated with ST09 for 24 h. Post incubation, MTT assay was performed to evaluate the cell viability in the 96-well plate and cells in the 6-well plate collected for protein.

### miRNA mimic transfection

miR-199a-5p mimic was purchased from Qiagen. For transfection, HEK293 at 70% confluency was transfected with 1 µg pmirGlo-DDR1-3’UTR and/or miR-199a-5p mimic (6.6 µM and 13.2 µM) in Opti-MEM I Reduced Serum Medium (Thermo Fisher Scientific) using Lipofectamine 2000 (Thermo Fisher Scientific). The firefly luciferase activity was measured post 48 h of transfection using the luciferase assay system (Promega) and bar graph plotted against the firefly luminescence.

### Western blot analysis

Briefly, for total protein extraction, control and drug-treated cells were harvested and lysed in RIPA buffer supplemented with Protease Inhibitor cocktail (Roche). For nuclear protein extraction, cells were lysed in 0.6% NP-40 Buffer (15 mM Tris, 15 mM NaCl, 60 mM KCl, 250 mM Sucrose, 5 mM MgCl_2_.6H_2_0, 1 mM CaCl_2_.2H_2_0, 1 mM DTT and 200 µM PMSF) centrifuged for 20 min at 15,000 rpm at 4°C. The pellet which is the nuclear extract were further lysed in RIPA buffer. The supernatant was taken as the cytoplasmic fraction. Protein concentration was determined by the Bradford method and 20–30 µg of protein was resolved by SDS-PAGE, followed by transfer onto PVDF membrane. Antibody concentrations used were BcL2 (Cloud Clone Corp.) 1:1000, Apaf1 (CST) 1:1000, Cytochrome C (CST) 1:500, Cleaved Caspase 9 (CST) 1:1000, Cleaved PARP (CST) 1:1000, MMP1 (Elabscience) 1:1000, MMP2 (Cloud Clone Corp.) 1:1000, MMP9 (Biolegend) 1:500, Vimentin (Elab science) 1:2000, Notch (CST) 1:500, Hes1 (CST) 1:1000, NFκB p65 (SCBT) 1:500, ERK1 (Cloud Clone Corp.) 1:1000, GAPDH (Cloud Clone Corp.) 1:8000, β-Tubulin (Cloud Clone Corp.) 1:5000, DDR1 (SCBT) 1:1000, Anti-rabbit IgG-HRP (CST) 1:5000 and Anti-rabbit IgG-HRP (CST) 1:5000. Based on the protein band of interest, the PVDF membrane was cut prior to antibody hybridisation according to the protein size. After the standard immunoblotting procedures, protein bands were detected using the enhanced chemiluminescence substrate (Thermo Fisher Scientific) and images acquired on ImageQuant LAS 4000 (GE Healthcare Life Sciences). Protein quantification was performed using ImageJ software (version 1.52a, https://imagej.nih.gov/ij/).

### Transwell migration assay

Pre-treated PA1 cells with ST09 drug for 48 h were seeded at a concentration of 1 × 10^5^ cells/well in serum-free media onto the inner chamber of the 24-well transwell plate with pore size 8.0 μm (Corning Inc.). The outer chamber of the transwell was filled with complete media containing serum, and the plate was incubated for 5 h. Post incubation, the media was removed, and the inner chamber was washed with 1X PBS to remove the non-migrated cells. The migrated cells were fixed in 70% Ethanol and stained using 0.2% Crystal Violet. Five different images were acquired per transwell, and percentage migrated cells were quantified using ImageJ software version 1.52a, (version 1.52a, https://imagej.nih.gov/ij/).

### Wound healing assay

PA1 cells were plated at a density of 1 × 10^5^ cells/well in a 24-well plate. After 48 h, wounds were created using a pipette tip, the scraped cells were removed with PBS wash. The cells were then incubated in 2% serum media with/without the ST09 drug. Images were obtained at different time points, the wound area was quantified manually using ImageJ software (version 1.52a, https://imagej.nih.gov/ij/) and percentage wound closure area plotted as a bar graph.

### Colony formation assay

PA1 cells were plated at a density of 1000 cells/well overnight, treated with desired concentrations of ST09 drug, and incubated further for 7 days. Post incubation, media was removed, the wells were washed with 1X PBS and stained with 0.2% Crystal Violet. The images were obtained and the colony area was quantified using ImageJ software version 1.52a, https://imagej.nih.gov/ij/) with the Colony Area plugin^21^.

### Immunofluorescence study

PA1 cells were plated on coverslips in a 6 well plate at a density of 1 × 10^5^ cells/well and treated with ST09 drug for 48 h. Post incubation the cells were washed, fixed in 4% paraformaldehyde and incubated for 15 min. The cells were then permeabilised with 0.5% Triton- X100 in PBS and blocked in 5% BSA for an hour. NFκB p65 (SCBT) was used at a concentration of 1:200 and incubated overnight at 4 °C. After washing with 1X PBS, goat anti-rabbit biotinylated antibody (1:200) (Novex) was added and incubated for 30 min at RT followed by Streptavidin-FITC conjugated antibody (1:200)(Sigma) for 30 min at RT. The coverslip was mounted with slow-fade antifade reagent (Thermo Fisher Scientific) and images captured using a fluorescent microscope.

### Real-time PCR

Total RNA from cells was extracted using RNAiso Plus (Takara). cDNA was synthesised with 2 µg RNA using PrimeScript RT Reagent Kit (Takara). Reaction mixtures were set in triplicates and run on StepOnePlus Real-Time PCR System (Applied Biosystems). The primer sequences used for this study are: 5S rRNA F: GCCTACAGCACCCGGTATTCC, 5S rRNA R: GTCTACGGCCATACCACCCTG, hsa-miR-199a-5p F: GGTCTCCCCAGTGTTCAGACTA, hsa-miR-199a-5p R: AGAAGGCGATTGATACGAGTCA, adapter : AAGGCGATTGATACGAGTCAGAACAGGTA, COX2 F: TCAGCCATACAGCAAATCC, COX2 R: TATACTGGTCAAATCCCACAC, 12S F: CAAACTGGGATTAGATACCC, 12S R: GAGGGTGACGGGCGGTGTGT.

### Ovarian tumor samples

HGSOC tumor samples and normal ovarian tissue samples were collected from Kidwai Memorial Institute of Oncology, Bangalore, India, with informed consent from the donors. The experimental protocols of this study were approved by the medical ethics committee of the hospital (Ref No. Kidwai Memorial Institute of Oncology/Medical Ethics Committee/006/Dec 2015) and all methods were carried out in accordance with relevant guidelines and regulations.

### Transcriptome and small RNA library preparation and sequencing

Ovarian tumor tissue and normal ovarian tissue samples were crushed in liquid nitrogen and RNA extracted using RNAiso Plus (Takara). For RNA isolation from ovarian cancer cell lines A2780 and PA1, cells treated with 40 nM ST09 for 48 h were used. After RNA quality checks, the transcriptome library was prepared using Illumina TruSeq RNA and miRNA library using miRNA Library Prep Kit v2. All the library samples were sequenced using Illumina Hiseq2500 to obtain 100 bp paired-end reads.

### Processing and analysis of sequenced samples

The raw fastq files were checked for quality using fastqc^22^. The bad-quality reads were trimmed using cutadapt^23^ and proceeded further for alignment. Raw reads were aligned to the hg38 reference genome using bowtie2^24^. The alignment output (SAM-sequence alignment map) was fed into samtools^25^ to get the binary file called BAM (binary alignment map). The BAM file was further used as an input to bed tools^26^ to calculate read counts. The read counts were further used to generate differentially expressed (DE) genes. The reads were normalised using RPKM (reads per kilobase per million). In the case of miRNA, alignment of sequences was done using the miRDeep2 tool^27^. miRDeep2 mapper aligns the miRNA sequencing file to the reference genome and miRDeep2 module maps onto the miRBase database for miRNA annotation. This tool gives known miRNA and novel miRNA files as output. The known miRNA file contains the expression counts, which were used for finding DE miRNAs. DESeq, an R package^28^, was used to obtain DE genes and miRNA. Significant genes and miRNAs were selected based on *p*-value and log2 fold change. The significant list of genes and miRNA was given as input to miRTarVis^29^ to obtain mRNA-miRNA interactions. These interactions were further analysed using miRmapper (R package) to get dominantly expressed miRNAs^30^. Graphs were plotted using GraphPad prism, and heatmap plotted using a pheatmap package in R^31^.

### Statistical analysis

Statistical analysis was performed using the GraphPad Prism tool (Version 5.03). One-way ANOVA followed by Tukey’s multiple comparisons was used to deduce the significance of different treated groups compared to its paired control. All results are represented as mean with standard deviation (SD). Significance was plotted based on *p* values, where *p* < 0.05 were considered significant. Significance was represented as *(*p*-value < 0.05), **(*p*-value < 0.01), ***(*p*-value < 0.001) and ****(*p*-value < 0.0001). Linear trend significancy was calculated using GraphPad Prism tool (Version 5.03) using one-way ANOVA followed by test for linear trend between mean and column number.

## Results

### ST09 drug inhibits cellular proliferation and induces apoptosis in ovarian cancer cell lines

**Figure 1:**
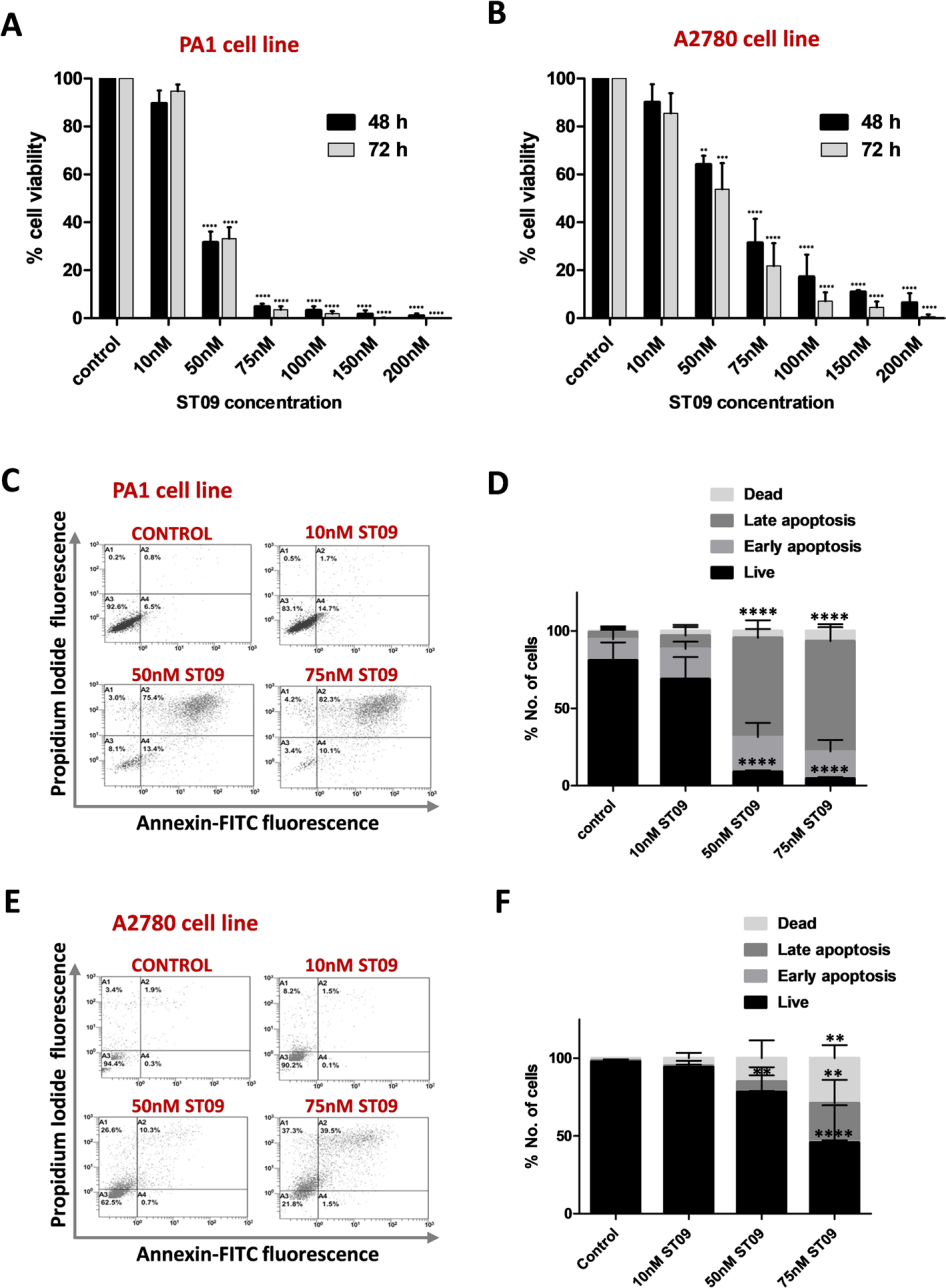
ST09 induced cytotoxicity and apoptosis in ovarian cancer cell lines. (**A**) MTT assay of PA1 cells treated with increasing concentration of ST09 for 48 and 72 h. (**B**) MTT assay of A2780 cells treated with increasing concentration of ST09 for 48 and 72 h. (**C)** Dot plot and (**D)** Quantification of AnnexinV-FITC/PI double staining of PA1 cells treated for 48 h with increasing concentrations of ST09 analysed by flow cytometry. **(E)** Dot plot and (**F)** Quantification of AnnexinV-FITC/PI double staining of A2780 cells treated for 48 h with increasing concentrations of ST09 analysed by flow cytometry. Flow cytometry quantification depicts cell percentage in each quadrant plotted as means ± SD from three independent experiments. The quadrants A1 represents healthy or live cells (AnnexinV- and PI-), A2 represents late apoptotic cells (AnnexinV + and PI +), A3 represents dead cells (AnnexinV- and PI +), and A4 represents early apoptotic cells (AnnexinV + and PI-). Flow cytometry dot plots were generated using Gallios software (version 1.2, https://www.mybeckman.in/flow-cytometry/software/kaluza-for-gallios). Significance was plotted based on *p* values and represented as **(*p*-value < 0.01), ***(*p*-value < 0.001) and ****(*p*-value < 0.0001). The post-test for linear trend was significant with *p*-value < 0.0001 for the MTT assays in both cell lines for both time points.

MTT assay was performed to study the cytotoxic effect of the curcumin derivative, ST09 in the ovarian cancer cell lines PA1 and A2780 cells. Increasing concentrations of ST09 were treated in both cell lines for 48 and 72 h. A significant time and dose-dependent effect could be observed in both cell lines at nanomolar concentrations (Fig. 1A,B), indicating its greater potency than its parent compound, which exerts its effect in micromolar concentrations^9,32^. The IC_50_ calculated for PA1 was 40 ± 2 nM, and A2780 was 53 ± 2 nM, based on MTT and LDH assays (Supplementary Fig. 1A,B).

AnnexinV-FITC/PI double staining assay was performed to investigate the mechanism of cell death induced by ST09. Both cell lines were treated with 10 nM, 50 nM, and 75 nM ST09 for 48 h, stained for AnnexinV-FITC/PI, and analysed by flow cytometry. The results indicated that at 50 nM ST09 concentration, 80% PA1 cells showed apoptotic cells compared to the 50% of A2780 (Fig. 1C–F), demonstrating PA1 to be more sensitive towards ST09 treatment. Necrotic cells represented only 3–8% in PA1, and 26.6% in A2780 at 50 nM ST09 concentration indicating ST09 induces apoptosis in these ovarian cancer cell lines.

### ST09 drug alters mitochondrial membrane potential and activates an intrinsic apoptotic pathway in ovarian cancer cell lines

**Figure 2:**
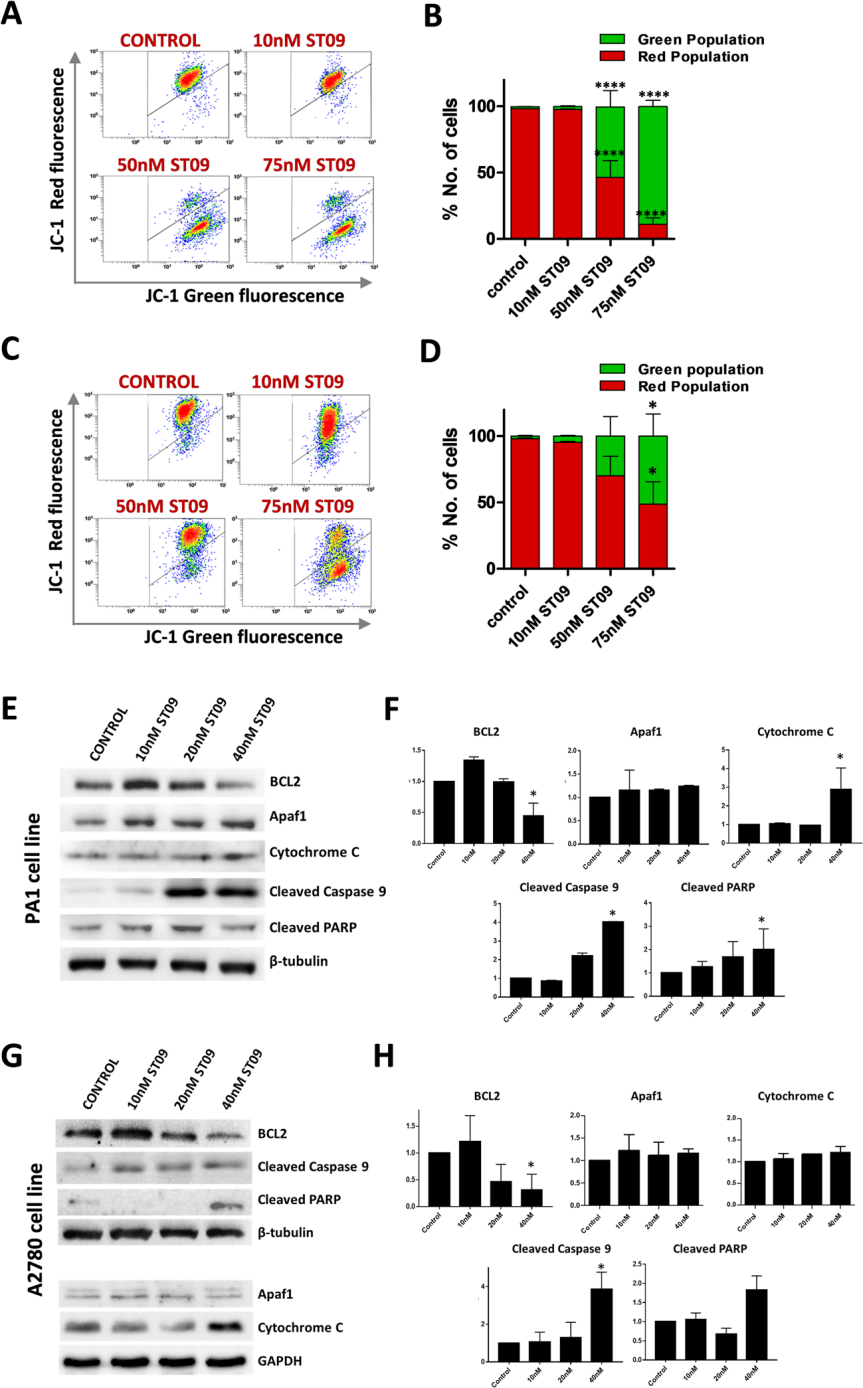
Altered mitochondrial membrane potential and expression of mitochondrial-mediated apoptotic proteins in ST09 treated ovarian cancer cell lines. (**A**) Dot plot and (**B**) Quantification of JC1 staining of PA1 cells treated for 48 h with increasing concentrations of ST09 analysed by flow cytometry. (**C)** Dot plot and (**D)** Quantification of JC1 staining of PA1 cells treated for 48 h with increasing concentrations of ST09 analysed by flow cytometry. Flow cytometry quantification percentage of cells with green JC-1 monomers (apoptotic cells) and red JC1 aggregates (healthy cells). The median fluorescence intensity (MFI) was plotted as means ± SD from three independent experiments. Flow cytometry dot plots are generated using Gallios software (version 1.2, https://www.mybeckman.in/flow-cytometry/software/kaluza-for-gallios). (**E)** Apoptotic protein expression and (**F)** Quantification of PA1 cells treated with increasing concentrations of ST09 for 48 h. (**G)** Apoptotic protein expression and (**H)** Quantification of A2780 cells treated with increasing concentrations of ST09 for 48 h. Uncropped gel images are provided in the supplementary information. Significance plotted based on *p* values, and represented as *(*p*-value < 0.05), and ****(*p*-value < 0.0001).

Cytotoxic drugs induce apoptosis via the intrinsic apoptotic pathway that involves the mitochondria^33^. For the induction of apoptosis, one of the initial cues is mitochondrial dysfunction which can be assessed by examining the mitochondrial transmembrane potential (MTP). Hence, the effect of ST09 treatment on mitochondrial function was detected using the cationic fluorescent dye JC1. Both ovarian cancer cell lines were treated with 10 nM, 50 nM, and 75 nM of ST09 drug for 48 h, stained with JC1, and subjected to flow cytometry analysis. As observed (Fig. 2A–D), increasing the concentration of ST09 in both ovarian cancer cell lines resulted in the shift of fluorescence spectra from red to green, indicating loss of MTP or dysfunctional mitochondria. This result suggests that ST09 induces cell death in both PA1 and A2780 ovarian cancer cell lines via mitochondrial-mediated apoptosis pathway.

Apoptosis is an orchestrated process where anti-apoptotic and pro-apoptotic factors aid in cytochrome c release from mitochondria and the apoptosome formation, which ultimately activates the caspase cascade leading to cell death. With the loss of MTP with ST09 drug treatment in both ovarian cancer cell lines, it was imperative to examine the expression of the regulatory proteins involved in this mitochondrial-mediated intrinsic apoptotic pathway. Thus, both ovarian cancer cell lines PA1 and A2780 were treated with 10, 20, and 40 nM of ST09 drug for 48 h and the expression of pro-survival factor BCL-2, pro-apoptotic proteins Bax and Bak, cytochrome C, apoptosome complex protease Apaf-1, and cleaved caspase 9 were evaluated. The expression of the 89 KDa catalytic fragment of cleaved PARP, which is an indicator for DNA repair impairment and a hallmark of apoptosis, was also evaluated. In both ovarian cancer cell lines (Fig. 2E–H), a significant downregulation of the anti-apoptotic BCL-2 and significant upregulation of cytochrome c and cleaved caspase 9 was observed, corroborating ST09 drug induces intrinsic apoptotic pathway in ovarian cancer cell lines.

### ST09 altered global gene expressions and upregulated miR-199a-5p in PA1 cells

**Figure 3:**
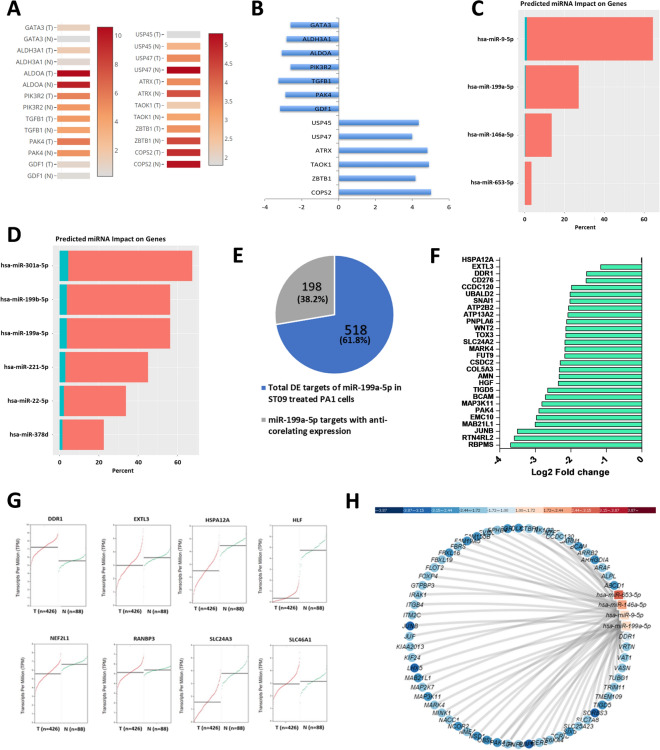
Induction of miR-199a-5p and identification of its targets in ST09 treated PA1 cells. (**A**) Heatmap of the DE genes from ST09 treated PA1 cells depicting their expression pattern in ovarian cancers obtained from GEPIA. (**B)** Bar graph depicting reversed expression of these genes upon ST09 treatment in PA1 cells compared to GEPIA ovarian cancer dataset (http://gepia.cancer-pku.cn/). (**C)** Bar plot obtained from miRmapper showing dominant miRNAs in ST09 treated PA1 cells with most impact on mRNA expression. (**D)** Bar plot obtained from miRmapper showing dominant miRNAs in ST09 treated A2780 cells with most impact on mRNA expression. The orange bar represents the percentage of targets, and the sea-green bar represents differentially expressed genes. (**E)** A pie chart representing the number of miR199a-5p targets differentially expressed in ST09 treated PA1 cells and the number of targets having an anti-correlating expression. (**F)** Bar plot showing the expression of miR199a targets in ST09 treated PA1 samples, where the x-axis is log2 Fold change, and the y-axis represents gene names. (**G)** Expression of shortlisted miR199a-5p targets in TCGA ovarian cancer database. (**H)** mRNA-miRNA interaction map of ST09 treated PA1 cells obtained from miRTarVis (gene names are listed in supplementary table).

To understand the global impact of ST09 induced alterations in gene expression pattern we performed transcriptome and miRNA sequencing of PA1 cells treated with 40 nM ST09. The sequencing reads, alignment percentage, PCA plot and DE mRNA/miRNAs are represented in Supplementary Figs. 2A–C and 3A,B. The top 200 significant differentially expressed (DE) genes were analysed for their status in ovarian cancer patients samples using GEPIA^34^ (Fig. 3A). We observed that 69% of the gene expression was restored to normal (when compared to tumour) upon ST09 treatment (Supplementary Table and Supplementary Fig. 4A). Figure 3B shows the representative genes where stemness genes (GATA3 and ALDH3A1), genes driving EMT and chemoresistance (PIK3R2, PAK4 and TGFB1) were downregulated and known tumour suppressors (ATRX and ZBTB1) were upregulated in ST09 treated PA1 cells. Upregulation of TAOK1, a regulator of apoptosis, was observed. Few deubiquitinases were activated which are low in ovarian tumour samples. Similar analysis was performed with ST09 treated A2780 cells where restoration of 25% of the genes to normal status was observed (Supplementary Table and Supplementary Fig. 4B–D). This analysis implies a global genetic shift induced by the ST09 drug towards a normal phenotype. We also subjected the common DE genes from both cell lines for pathway analysis and found pathways like ECM-receptor interaction pathway and pluripotency to be regulated by ST09 (Supplementary Table and Supplementary Fig. 4E,F).

Next, we sought to identify the involvement of miRNAs in the ST09 altered gene expressions. We used mirTarVis to identify all anti-correlating genes and miRmapper to identify dominant miRNAs with the most significant impact on the mRNAs. Of all the DE miRNAs, miRmapper identified four dominant miRNAs in ST09 treated PA1 cells: miR-9-5p, miR-199a-5p, miR-146a-5p, and miR-653-5p (Fig. 3C), and six dominant miRNAs in ST09 treated A2780 cells: miR-301a-5p, miR-199a-5p, miR-199b-5p, miR-221-5p, miR-22-5p, and miR-378d (Fig. 3D). miR-199a-5p came up common among both ovarian cancer cell lines but interestingly, PA1 and A2780 showed upregulation and downregulation of miR-199a-5p, respectively. Analysis of the ovarian cancer data from public repositories showed low levels of mir-199a-5p in ovarian cancer^35^. To understand cell type specific regulation of the genes and miRNA leading to alterations in the phenotype, miR-199a-5p was selected for further investigation. Targetscan identified 38.8% genes to have an anti-correlative expression with miR-199a-5p in the ST09 treated PA1 cells and the anti-correlating genes in log fold change are depicted in Fig. 3E,F.

Towards identifying the miR-199a-5p targets, from the list of miR-199a-5p anti-correlating genes, we filtered genes with 5 or more target sites at its 3’UTR site using miRmap (Supplementary Fig. 2D). To further narrow down to the most relevant anti correlating gene pair, we analyzed the expression of these genes in the TCGA ovarian cancer database. Interestingly, except for DDR1 all the other genes were downregulated in tumors demonstrating no correlation with miR199a-5p expression Fig. 3G. miRTarVis identified DDR1 to be one of the miR-199a-5p targets in the ST09 treated PA1 cells (Fig. 3H). In ST09 treated A2780 cells the DDR1 expression was marginally high and anti-correlating to miR199a-5p expression. Thus, due to the negative correlation between DDR1 and miR-199a-5p in both cell lines, we pursued DDR1 for our further studies.

### DDR1, a direct target of miR-199a-5p

**Figure 4:**
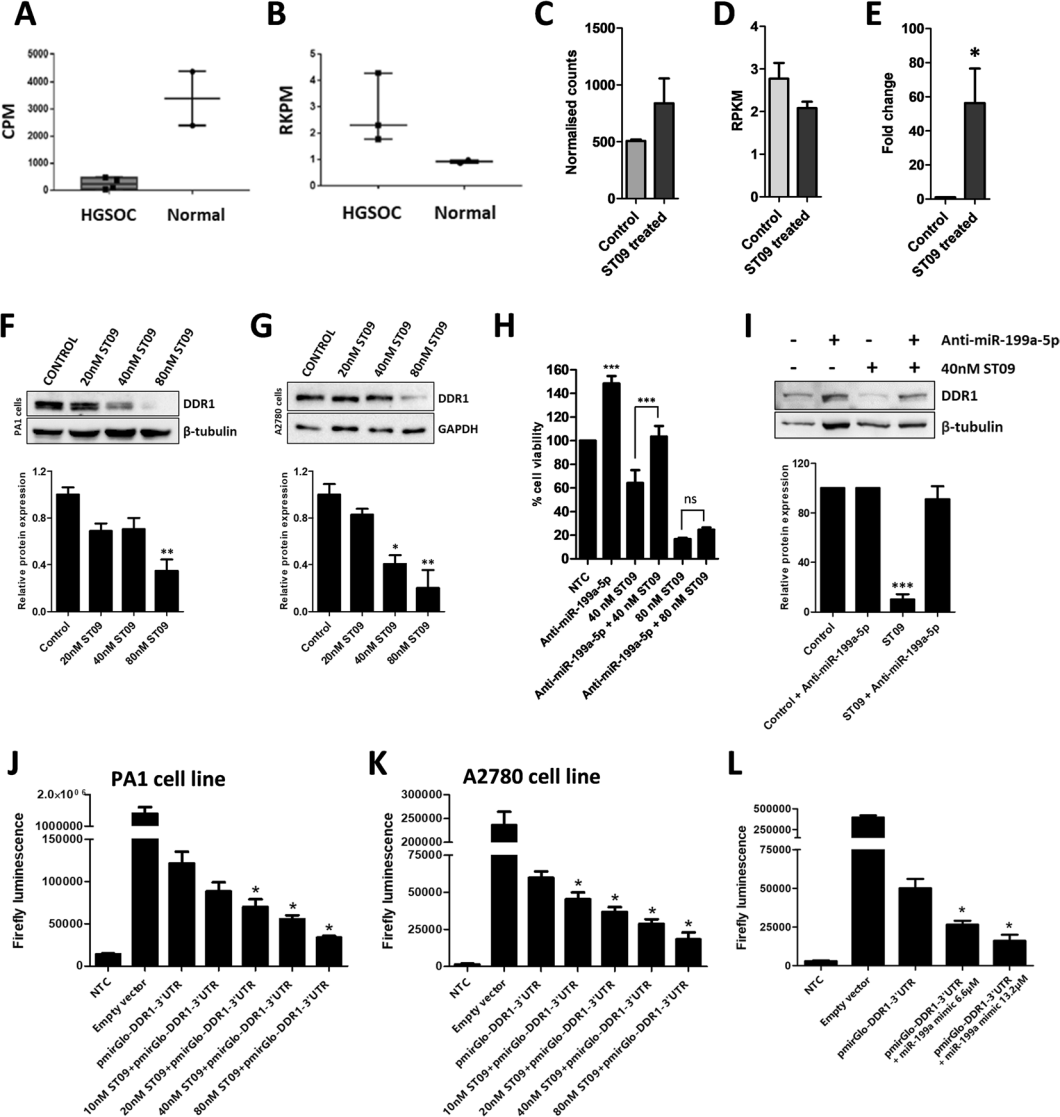
Negative correlation of DDR1 and miR-199a-5p in ovarian cancers and DDR1 is a direct target of miR-199a-5p. (**A**) mir-199a-5p expression in Indian HGSOC samples. (**B)** DDR1 expression in Indian HGSOC samples. (**C)** Bar graph depicting miR-199a-5p expression in ST09 treated PA1 cells where y-axis represents normalised counts obtained from miRDeep2 tool (**D)** Bar graph depicting DDR1 expression (in RPKM) in ST09 treated PA1 cells. (**E)** Represents mir-199a-5p expression in 40 nM ST09 treated PA1 cells by qPCR. (**F)** Expression and quantification of DDR1 protein in PA1 cells treated with increasing concentrations of ST09 for 48 h. (**G)** Expression and quantification of DDR1 protein in PA1 cells treated with increasing concentrations of ST09 for 48 h. (**H)** MTT assay of PA1 cells transfected with/without anti-miR-199a-5p (2.5 nM) and ST09. (**I)** Expression and quantification of DDR1 protein in PA1 cells transfected with/without anti-miR-199a-5p and 40 nM ST09. (**J)** Bar graph quantification of luciferase activity upon transfecting PA1 cells with pmirGlo-DDR1-3’UTR with increasing concentrations of ST09. (**K)** Bar graph quantification of luciferase activity upon transfecting A2780 cells with pmirGlo-DDR1-3’UTR with increasing concentrations of ST09. (**L)** Bar graph quantification of luciferase activity upon transfecting HEK293 cells with pmirGlo-DDR1-3’UTR in combination with miR-199a-5p mimic (6.6 and 13.2 µM). The luciferase activity is represented as the relative fluorescence intensity of firefly luminescence. Uncropped gel images are provided in the supplementary information. All bar graphs are plotted as mean ± SD from three independent experiments. Significance was plotted based on *p* values, and represented as *(*p*-value < 0.05), **(*p*-value < 0.01), and ***(*p*-value < 0.001).

DDR1 is reported to be highly expressed in various cancer types^36^. To analyse its expression in ovarian cancers and its inverse correlation with miR-199a-5p, we examined their expression pattern in HGSOC tumor samples obtained from Indian patients. Three stage III HGSOC tumor samples and two normal ovarian tissue samples were obtained for this study. Transcriptome and miRNA analysis of these samples corroborated the high DDR1 and low miR-199a-5p expression, establishing the anti-correlative pattern of DDR1 and miR-199a-5p in ovarian cancers (Fig. 4A,B). The sequencing reads, alignment percentage, PCA plot and anti-correlating mRNA-miRNA pairs are represented in Supplementary Fig. 5A–C.

Figure 4C,D represents the expression and inverse correlation of DDR1 and miR-199a-5p obtained from the mRNA-miRNA analysis of ST09 treated PA1 cells (Fig. 4C,D). qPCR analysis of miR-199a-5p in ST09 treated PA1 cells showed significant upregulated expression (Fig. 4E). With the upregulated expression of miR-199a-5p in ST09 treated PA1 cells, and its inverse correlation with DDR1 in ovarian cancers, we examined the protein expression of DDR1 in both ST09 treated PA1 and A2780 ovarian cancer cell lines. DDR1 expression showed significant dose-dependent downregulation upon ST09 treatment (Fig. 4F,G). Although transcriptomic analysis showed DDR1 upregulation in A2780, at protein level it was downregulated. To investigate the difference between the protein and RNA, we analysed miRNA targets which might regulate DDR1 in A2780 at translational level. Of 14 miRNA which were upregulated and found as targets of DDR1 50% were upregulated, indicating cell type specific regulation of gene expression. To understand the functional consequence of miR-199a-5p and its interaction with DDR1, we transfected PA1 cells with anti-miR-199a-5p, a synthetic miRNA inhibitor and observed anti-miR-199a-5p alone increased cellular proliferation, and this reduced proliferation induced by ST09 was restored with anti-miR-199a-5p treatment (Fig. 4H). A higher dose of 80 nM ST09 had no effect in activating proliferation. We also observed that the ST09 induced downregulation of DDR1 protein level was restored with anti-miR-199a-5p, suggesting that endogenous miR-199a-5p binding to DDR1 was abrogated (Fig. 4I).

To validate the direct interaction of miR-199a-5p with the 3′ UTR of DDR1, luciferase assay was performed by transfection of pmirGlo-DDR1-3’UTR into both ST09 treated ovarian cancer cells. The assay showed a significant dose-dependent decrease of luciferase activity in both cells (Fig. 4J,K) indicating the induction of endogenous miR-199a-5p upon ST09 treatment. To further validate this interaction, HEK293 cells co-transfected with miR-199a-5p mimic at 6.6 and 13.2 µM showed a marked decrease in luciferase activity (Fig. 4L). Taken together, these results indicate that miR-199a-5p directly binds to 3’UTR of DDR1 mRNA, decreasing its expression and in ST09 treated ovarian cancer cell lines, miR-199a-5p mediates the downregulation of DDR1.

### ST09-induced DDR1 downregulation inhibits MMPs, affecting migration, wound closure, and colony-forming properties in PA1 cells

**Figure 5:**
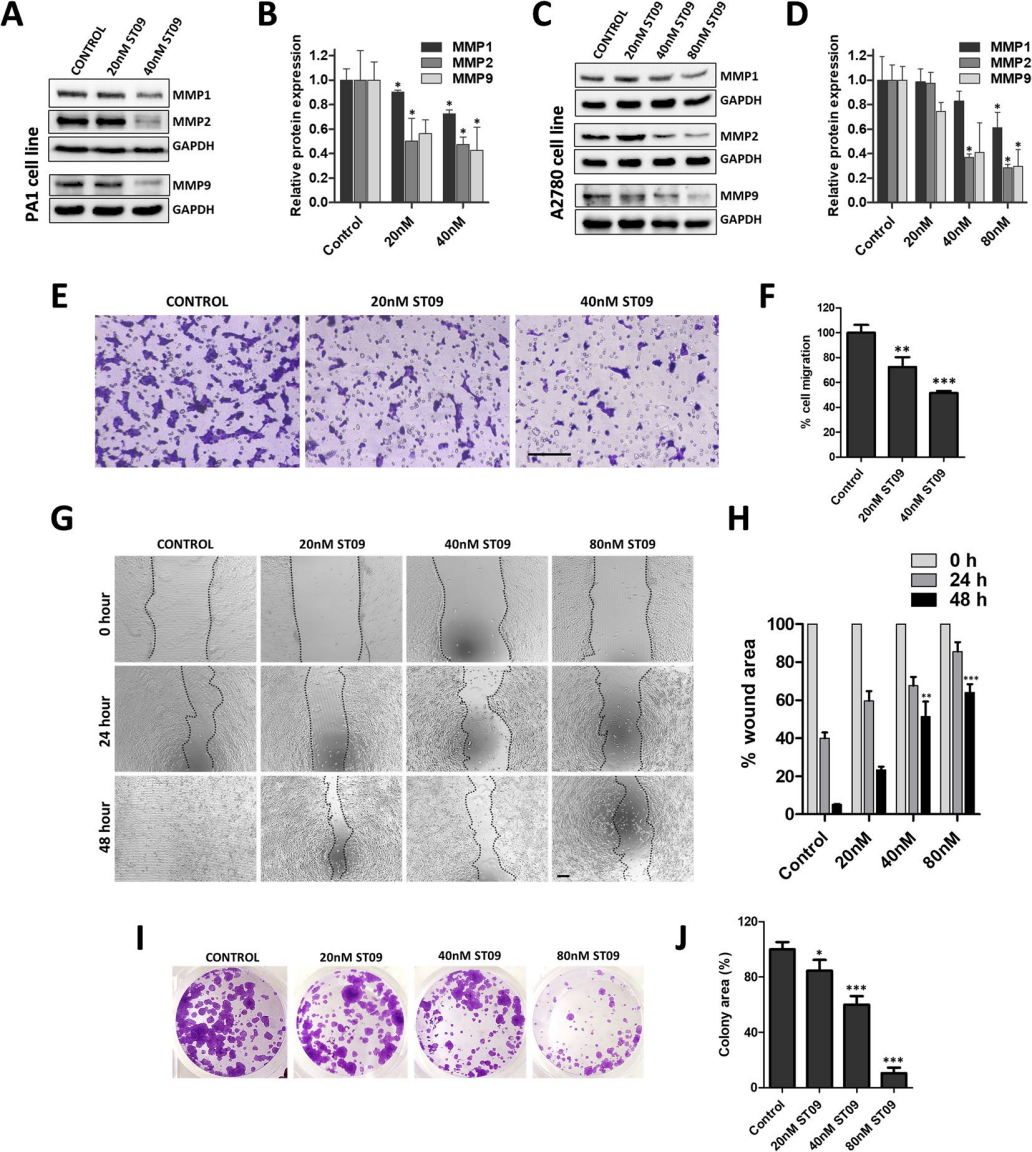
ST09 downregulates downstream DDR1 target, MMPs inhibiting migration, wound closure and colony-forming properties in PA1 cells. (**A**) Expression of MMP proteins and (**B)** corresponding quantification in PA1 cells treated with increasing concentrations of ST09 for 48 h. (**C)** Expression of MMP proteins and (**D)** Corresponding quantification of A2780 cells treated with increasing concentrations of ST09 for 48 h. (**E)** Migration assay images and (**F)** corresponding quantification of the percentage migrated ST09-treated PA1 cells. Scale bar represents 200 µm (**G)** Wound healing assay images and (**H)** corresponding quantification representing percentage wound area at 0, 24 and 48 h time points of PA1 cells treated with increasing concentrations of ST09. Scale bar represents 200 µm (**I)** Clonogenic assay images and (**J)** corresponding quantification representing colony area percentage of PA1 cells treated with increasing concentrations of ST09. Uncropped gel images are provided in the supplementary information. All bar graphs are plotted as mean ± SD from three independent experiments. Significance was plotted based on *p* values, and represented as *(*p*-value < 0.05), **(*p*-value < 0.01), ***(*p*-value < 0.001) and ****(*p*-value < 0.0001).

DDR1 is associated with tumor progression and is linked to regulating cell adhesion and migration via modulating the expression of MMPs like MMP1, MMP2, and MMP9^37,38^. MMPs are regulatory proteases involved in the degradation of the ECM, thereby promoting cancer progression, invasion, and metastasis. Thus, as a consequence of DDR1 downregulation upon ST09 treatment, we evaluated the expressions of these MMPs in both ovarian cancer cell lines. The expressions of MMP1, MMP2, and MMP9 showed significant downregulation upon ST09 treatment in both PA1 (Fig. 5A,B) and A2780 (Fig. 5C,D) cells suggesting DDR1 signaling might be one of the factors responsible for the observed change in MMPs.

With MMP expressions downregulated upon ST09, it was imperative to examine the migratory capacity of these cells, as it is a measure of cancer cell’s ability to invade and metastasize. We performed transwell migration to assess an individual cell's capacity to migrate and wound healing assay to assess the collective migratory capacity. PA1 ovarian cancer cells were used to perform these assays. They grow in monolayers compared to A2780, which tend to grow in clusters. Transwell migration assay where the serum-containing media was used as a chemoattractant showed a significant dose-dependent reduction in the migratory capacity of PA1 cells with a 50% reduction at 40 nM ST09 treatment (Fig. 5E,F). Likewise in the wound healing assay, compared to control where the wounds were cleared in 48 h, the ST09 treated cells showed no wound closure with dose-dependent increase in the wound area (Fig. 5G,H).

A clonogenic assay was performed to assess the self-renewal, survival, and clonal expansion of single PA1 cells under the effect of the ST09 drug. The assay showed a significant dose-dependent decrease in the number of colonies upon ST09 treatment (Fig. 5I,J). Collectively, these studies demonstrate the migrastatic property of ST09 in the ovarian cancer cell lines.

### ST09 targets downstream DDR1 pathways exerting anti-metastatic and anti-invasive properties in ovarian cancer cells

**Figure 6:**
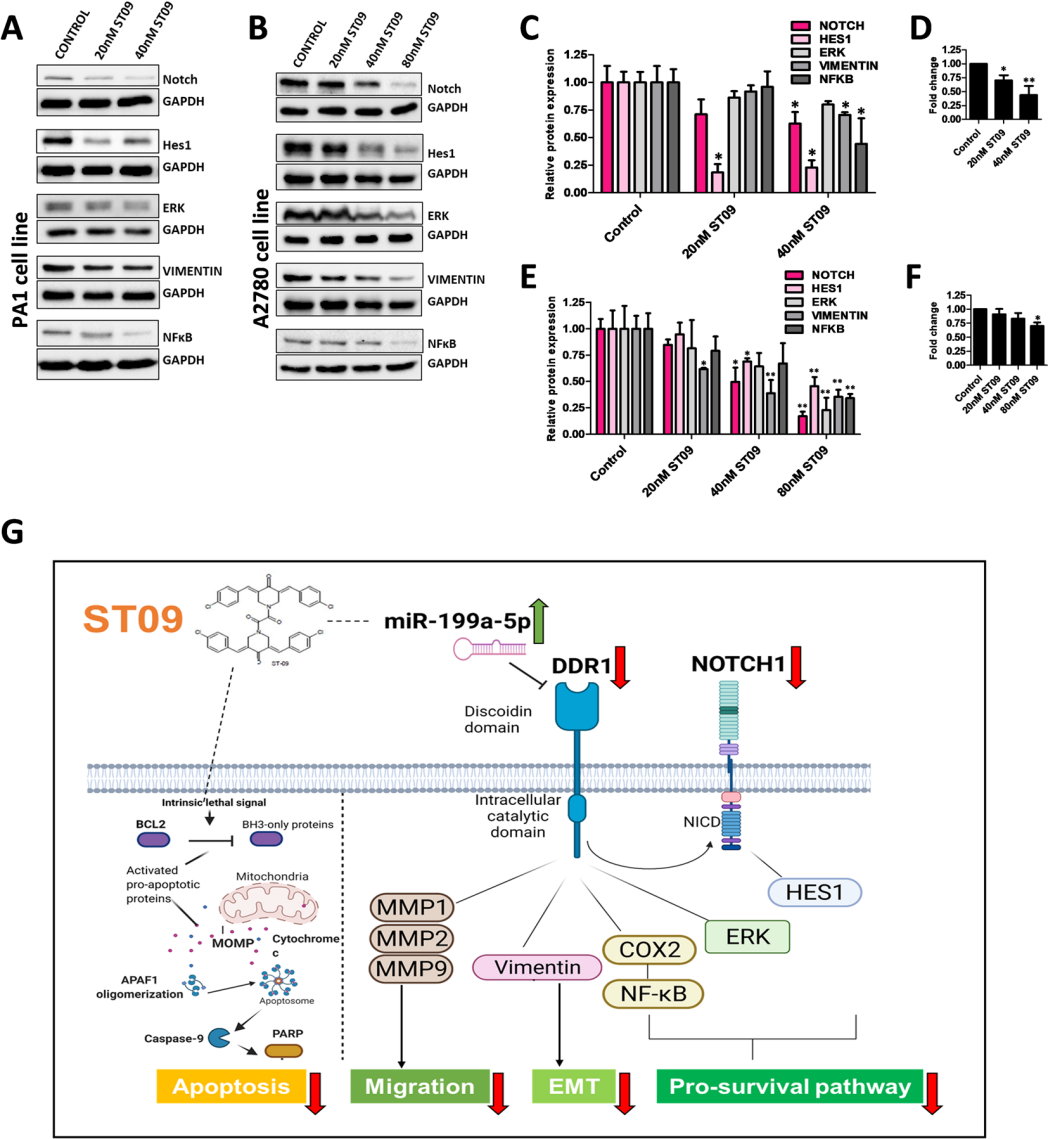
ST09 downregulates multiple downstream DDR1 pathways in ovarian cancer cell lines. (**A**) Protein expression of NOTCH, HES1, ERK, Vimentin and NF-κB, and (**C)** corresponding protein quantifications in PA1 cells treated with increasing concentrations of ST09 for 48 h. (**B)** Protein expression of NOTCH, Hes1, ERK, Vimentin and NF-κB, and (**D)** corresponding protein quantifications in A2780 cells treated with increasing concentrations of ST09 for 48 h. (**E)** COX2 transcript levels in ST09 treated PA1 cells (**F)** COX2 transcript levels in ST09 treated A2780 cells. All bar graphs are plotted as mean ± SD from three independent experiments. Uncropped gel images are provided in the supplementary information. (**G)** Graphical representation of the effects of ST09 in ovarian cancer cells (created with BioRender.com). Significance was plotted based on *p* values, and represented as *(*p*-value < 0.05), **(*p*-value < 0.01), ***(*p*-value < 0.001) and ****(*p*-value < 0.0001).

DDR1 is linked to activating several intracellular signaling cascades involved in survival, proliferation, differentiation, and invasion like the ERK, NOTCH and NFκB signaling pathways via its downstream receptor kinase activity^13,39^. Likewise, DDR1 is also reported to mediate epithelial-to-mesenchymal transition (EMT), aiding in cell motility and invasiveness^40^. All these pathways drive the metastatic and invasive characteristics of the tumor, aiding in cancer progression. Therefore, to understand the ST09 mediated effects on the downstream DDR1 pathways, we analysed the expression of these mediators in both PA1 and A2780 ovarian cancer cell lines treated with increasing concentrations of ST09 for 48 h. We observed significant dose-dependent downregulation of protein levels of ERK, NOTCH, and its downstream regulator HES1 and an EMT marker Vimentin (Fig. 6A–C,E). NF-κB protein levels and its downstream target COX2 transcript levels also showed a significant downregulation (Fig. 6A–F). Immunofluorescence study and protein expression in nuclear extracts of ST09 treated PA1 cells showed downregulated expression pattern of NF-κB further corroborating its decreased translocation to nuclei **(**Supplementary Fig. 6A–C). Taken together, these results indicate that ST09 induced DDR1 downregulation along with its downstream targets confers an anti-invasive and anti-metastatic characteristic in the ovarian cancer cell lines PA1 and A2780. Figure 6G represents the overall effect of ST09, the curcumin derivative on ovarian cancer cell lines in inducing apoptosis and migrastatic properties.

## Discussion

Around 60% of chemotherapeutic drugs used for cancer treatment are synthetic derivatives of natural compounds^41^. The natural phytochemical curcumin has immense potential in treating various diseases other than just cancers, due to its multi-modal activity with multiple cellular targets resulting in numerous biological effects^3^. Numerous curcumin analogs, derivatives, and even novel drug delivery systems have been developed to improve its efficacy. However, none have entered clinical trials yet^42^.

Here, we show the curcumin derivative ST09 effective against ovarian cancer cell lines A2780 and PA1 cells featuring it as a potential drug for ovarian cancer therapy. Notably, ST09 induces cytotoxicity at nanomolar ranges compared to its parent compound curcumin or other curcumin derivatives reported to date, which acts at micromolar concentrations^43^. Moreover, we have previously reported that ST09 functions only against tumorigenic cells but not on normal cells^10^. ST09 induces cytotoxicity in ovarian cancer cell lines triggering apoptosis as observed by Annexin V-FITC/PI staining. Further investigations proved mitochondria-mediated apoptotic pathways activated with mitochondrial membrane depolarization and induction of anti-apoptotic Bax, cytochrome c release, caspase activation, and downregulation of pro-apoptotic proteins like BCL2. Overall, a dose dependent effect of the drug ST09 could be observed in both the ovarian cancer cell lines. Of significance is its substantial impact on the undifferentiated stem-cell like PA1 cell line compared to A2780, which is more of a differentiated ovarian cancer cell line.

Global gene expression pattern upon ST09 treatment in the ovarian cancer cells revealed a reversed gene expression pattern from cancerous to normal phenotype. Stemness associated genes are involved in the induction of drug resistance and self-renewal via cancer stem cells^44^. Transcriptome sequencing of ST09 treated PA1 and A2780 cells depicted reversal of stemness, proliferation and EMT related genes, suggesting ST09 to be involved in inducing a shift in the pro-oncogenic: anti-oncogenic signals. The small non-coding microRNAs involved in post-transcriptional regulation of gene expression have recently gained immense significance in cancer biology, providing cues on cancer progression, invasion, metastasis, and drug sensitivity^45^. Integrated microRNA and transcriptome analysis in the ovarian cancer cell lines post ST09 drug treatment identified miR-199a-5p to be aberrantly expressed and as one of the dominantly expressed miRNAs. miR-199a-5p has been previously implicated in colorectal cancers, gliomas, breast cancers, squamous cell carcinomas of the cervix, bladder cancer, and hepatocellular carcinoma to function as a tumor suppressor but in certain other cancer types like melanoma and gastric cancer to function as an oncomir^46^. The dichotomic function of miRNAs in cancers is a tissue-specific functionality^47^.

Further, miRNA-mRNA interaction analysis identified DDR1, a non-integrin collagen receptor, as a target of miR-199a-5p that was downregulated upon the ST09 drug. ST09 treated PA1 and A2780 cells showed a dose-dependent decrease of DDR1 protein levels and upregulation of miR-199a-5p. A loss-of-function experiment with transfection of anti-miR-199a-5p abrogated the ST09-induced downregulation of DDR1, indicating inactivation of ST09-induced endogenous miR-199a-5p. The luciferase reporter assay confirmed DDR1 as a direct target of miR-199a-5p in both ST09 treated ovarian cancer cells. In A2780 cells, though the transcript level did not complement the protein expression levels upon ST09 treatment, further investigations revealed many other DDR1 targeting miRNAs to be upregulated from the miRNA sequence analysis suggesting cell type specific interaction which needs to be further investigated. miR-199a-5p:DDR1 interaction was further validated by a gain-of-function study using luciferase reporter assay in HEK293 cells co-transfected with miR-199a-5p mimic. This miR-199a-5p: DDR1 inverse correlation was previously reported in other cancer types like colorectal cancer, hepatocellular carcinoma, and breast cancer, but not in ovarian cancers^48–50^. This interaction was associated with increased tumor invasion with poor prognosis in these cancer types. The inverse correlation of mir-199a-5p with DDR1 was also validated in stage III Indian HGSOC tumor samples with normal ovarian tissue where DDR1 gain with miR-199a-5p loss was observed. This association in this study provides the mechanistic basis of DDR1 regulation by miR-199a-5p upon ST09 treatment.

DDR1 is associated with tumor progression, invasion, and drug resistance^13,37^. Elevated DDR1 levels in high-grade and advanced ovarian cancers has been implicated with poor survival^51^. Knockdown of DDR1 in ovarian cancer cells increased its sensitivity towards cisplatin^52^. Likewise, DDR1 depleted breast cancer cells exhibited increased sensitivity towards etoposide^53^. DDR1 has been an attractive target for treating metastatic cancers like colorectal cancer, breast cancer, gastric cancer, pancreatic cancer, and lung cancer^54^. It is a collagen receptor with RTK activity regulating numerous downstream signaling pathways like differentiation, wound healing, migration, survival, and proliferation. Specifically, DDR1 is reported to activate MMPs and migration in various cancers^55,56^. In this study, both ST09 treated ovarian cancer cell lines showed downregulation of MMP1, MMP2, and MMP9 with DDR1 loss. Moreover, the single and collective cell migratory capacity of the ST09-treated PA1 cells was markedly reduced. Survival capability assessed by clonogenic assay also revealed ST09 cytotoxicity in PA1 cells. These studies point out that ST09 induces migrastatic properties, possibly involving the downstream DDR1 pathways.

DDR1 regulates ERK, NOTCH, and EMT pathways, which are all involved in tumor invasion and metastatic dissemination^57,58^. EMT is considered to be one of the pivotal steps in metastasis along with activation of migration. A marker for EMT, the mesenchymal protein vimentin, showed a significant downregulation upon ST09 treatment in both cell lines. The ST09-induced DDR1 loss also resulted in the downregulation of ERK and NOTCH proteins implying ST09 ability to inhibit these pathways. DDR1 is reported to regulate chemoresistance via activation of the COX2-mediated NF-κB prosurvival pathway^53^. Interestingly, NF-κB was also reported to be a direct target of miR-199a-5p and inversely correlated in ovarian cancer cells^59^. The ST09-treated ovarian cancer lines showed decreased NF-κB expression and COX2 transcript levels, indicating the miR-199a-5p: DDR1 axis to be involved. We also observe nuclear NF-κB expression to be down regulated in a dose dependent manner. Interestingly, NF-kB levels at the transcriptome level did not fall either in a significantly downregulated or upregulated categories, indicating translational repression rather than mRNA degradation as a mechanism of miRNA action.

## Conclusion

The curcumin derivative ST09 induces cytotoxicity in ovarian cancer cell lines PA1 and A2780, triggering intrinsic mitochondrial apoptotic pathways. The miRNA-transcriptome profiling in these ovarian cancer cell lines treated with the ST09 drug indicated the miR-199a-5p/DDR1 axis to be involved in tumor-suppressive functions. We report the inverse correlation of miR-199a-5p: DDR1 in ovarian cancers confirmed with patient cohort analysis. ST09 induced down-regulation of DDR1, a receptor tyrosine kinase and its downstream pathways like MMP activation, migration, and EMT. The findings of this study demonstrate that the ST09 stimulates anti-survival pathways indicating a chemosensitive nature of the drug with migrastatic properties and is a potential chemotherapeutic candidate for ovarian cancer treatment.

## Supplementary Information

Supplementary Figures.

Supplementary Tables.

## Data Availability

The data that support the findings of this study are available from the corresponding author on reasonable request.

## Abbreviations

DDR1: Discoidin domain receptor 1
MMP: Matrix metalloproteinase
HGSOC: High-grade serous ovarian cancer
EMT: Epithelial-to-mesenchymal transition
BCL2: B-cell lymphoma 2
APAF1: Apoptotic protease activating factor 1
EAC: Ehrlich ascites carcinoma
