# Supplementary material for: Curcumin derivative ST09 modulates the miR-199a-5p/DDR1 axis and regulates proliferation and migration in ovarian cancer cells
Source: Sci Rep. 2021 Nov 26;11:23025. doi: 10.1038/s41598-021-02454-1 (PMC8626492; doi:10.1038/s41598-021-02454-1)

**Curcumin derivative ST09 modulates the miR-199a-5p/DDR1 axis and regulates proliferation and migration in ovarian cancer cells**

Febina Ravindran^1^, Jinsha Koroth^1,2^, Meghana Manjunath^1,2^, Suchitra Narayan^1^, Bibha Choudhary^1,*^

^1^ *Institute of Bioinformatics and Applied Biotechnology, Electronic city phase 1, Bangalore, India*

^2^ *Manipal Academy of Higher Education, Manipal, India*

^*^ *Corresponding author: Bibha Choudhary, Institute of Bioinformatics and Applied Biotechnology, Electronic city phase 1, Bangalore, Karnataka, India, Tel: 080-28528900, E-mail:* [*vibha@ibab.ac.in*](mailto:vibha@ibab.ac.in)


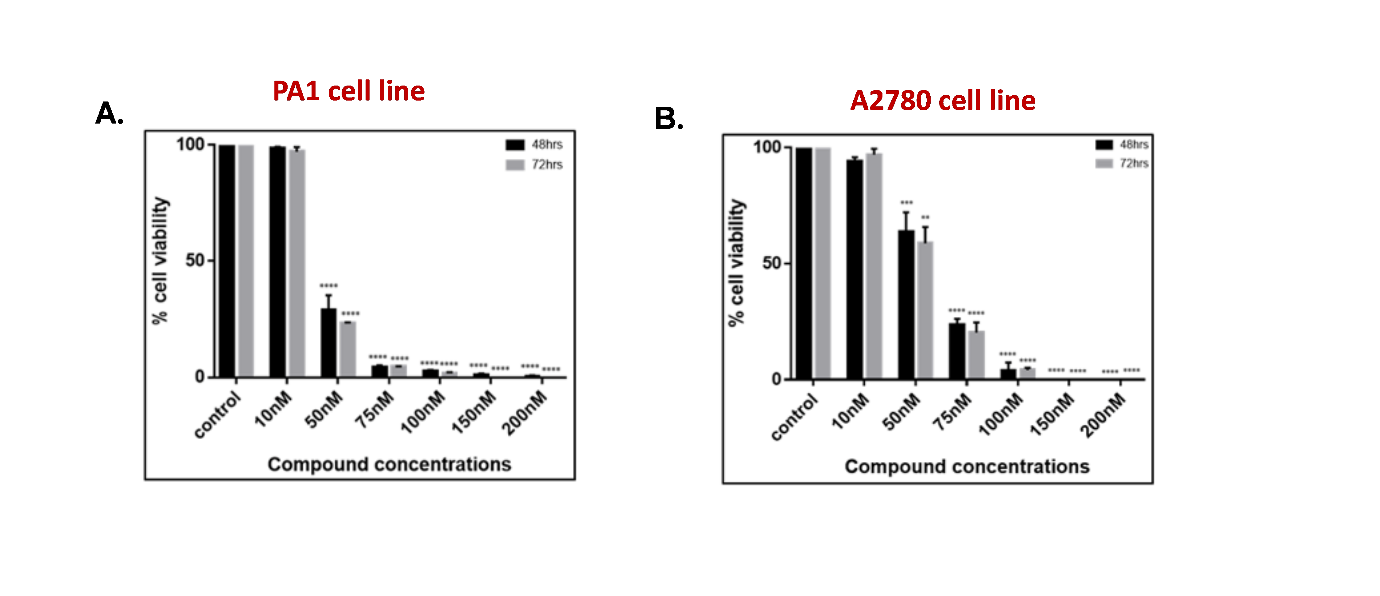


**Supplementary Figure 1: LDH assay of ST09 treated ovarian cancer cell lines**. **A** LDH assay of PA1 cells treated with increasing concentration of ST09 for 48 and 72 hours. **B** LDH assay of A2780 cells treated with increasing concentration of ST09 for 48 and 72 hours. Significance plotted based on p values, and represented as ** (p-value < 0.01), *** (p-value < 0.001) and **** (p-value < 0.0001).


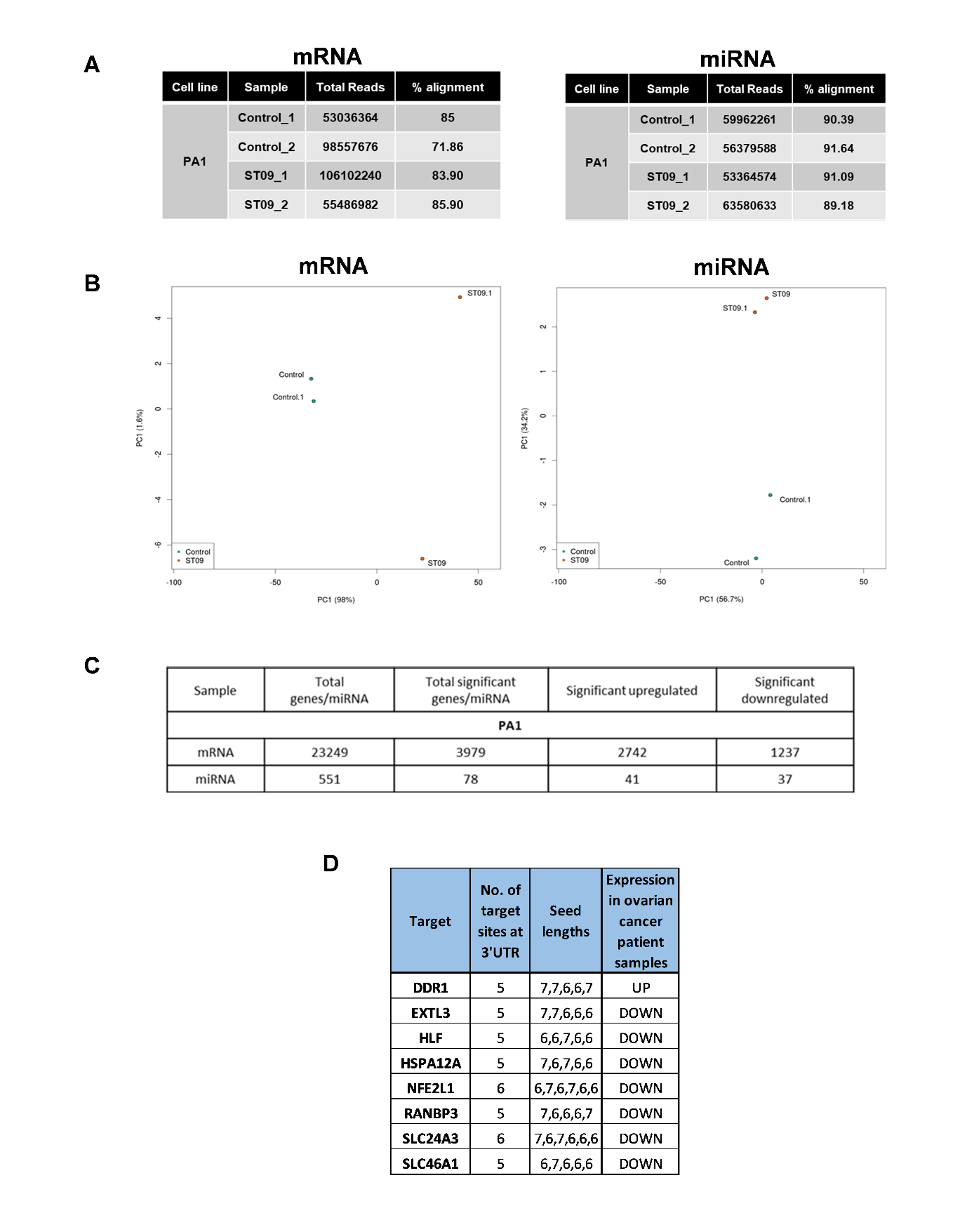


**Supplementary Figure 2: A** Table representing number of reads and alignment percentage for mRNA and miRNA in the ST09 treated PA1. **B**. PCA plot for mRNA and miRNA in the ST09 treated PA1 cells. Control samples are shown in green colour and orange colour represents ST09 treated samples. **C** Table showing total genes/miRNA and number of significant genes/miRNAs in ST09 treated PA1 cells **D** Targets of miR199a-5p having more than 5 targets sites at its 3’UTR


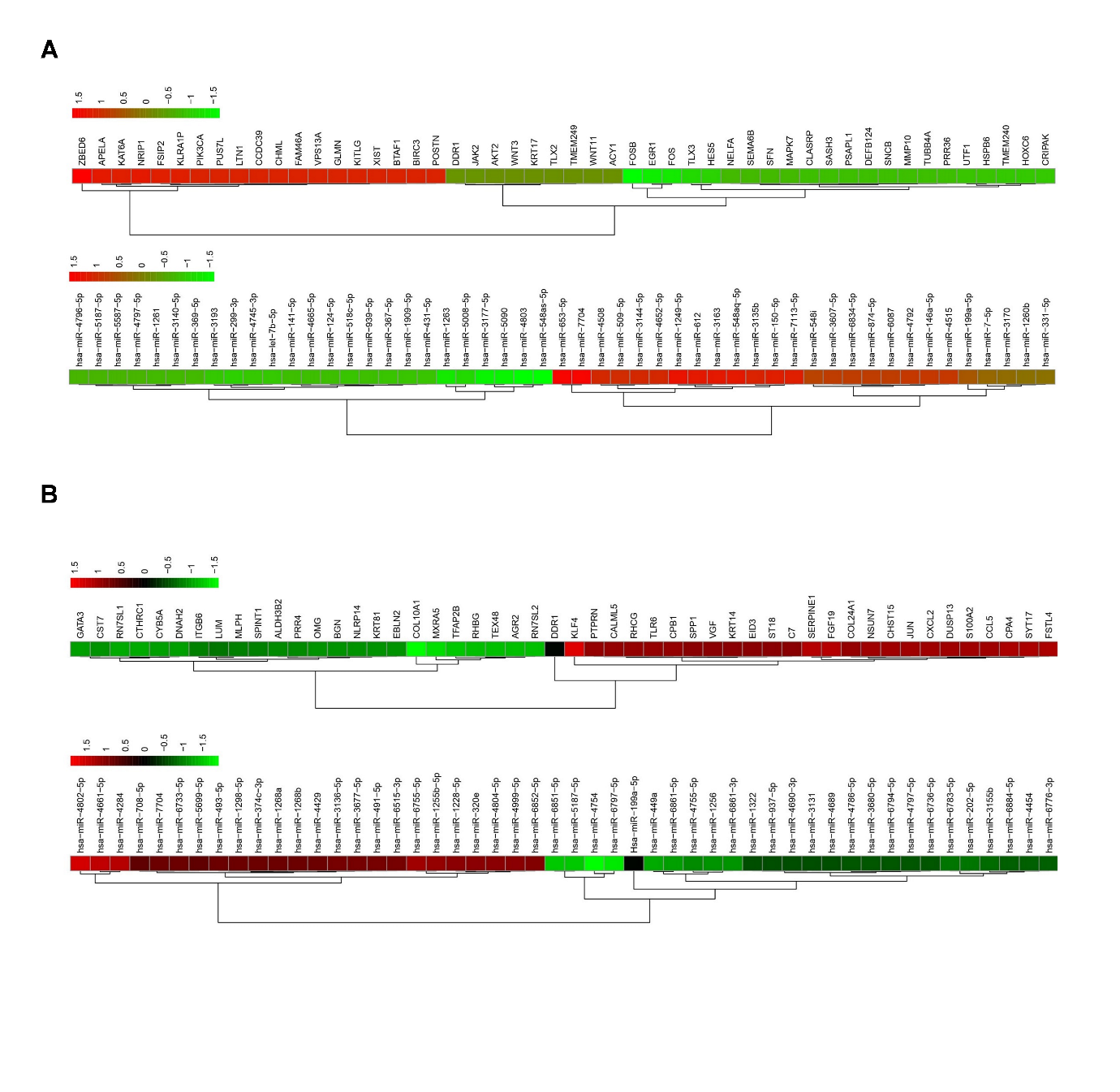


**Supplementary Figure 3: A** Heatmap of Top 50 significant differentially expressed mRNA and miRNA in ST09 treated PA1. **B** Heatmap of Top 50 significant differentially expressed mRNA and miRNA in ST09 treated A2780. Significant cut-off used is p-value < 0.05 and log2 fold change greater or less than 1.5 for mRNA and p-value < 0.05, log2 fold change 1 for miRNA. In the heatmap, red colour is used to depict upregulated genes/miRNA and green colour for down regulated genes/miRNA. The scale used for the heatmap is column.


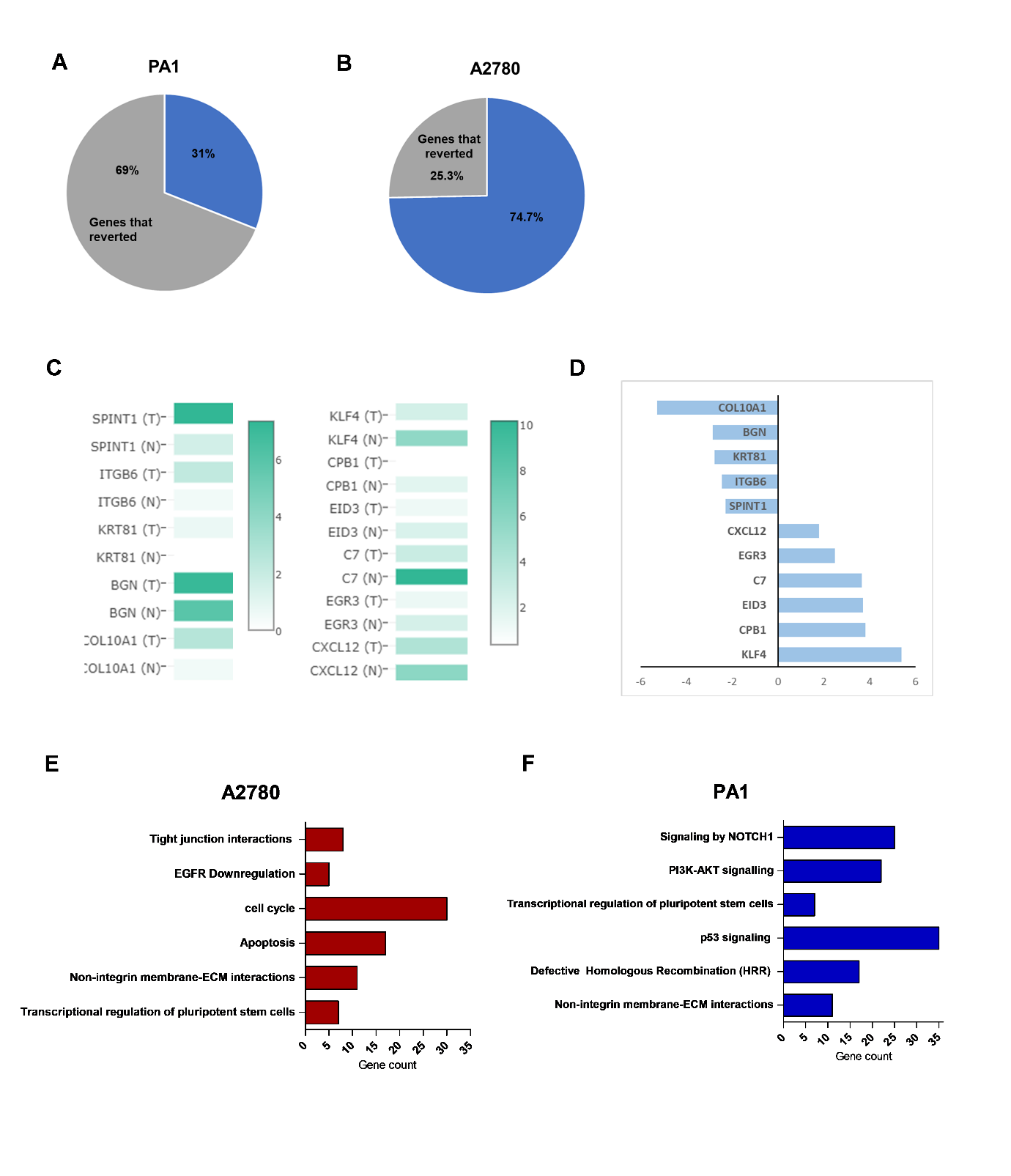


**Supplementary Figure 4:** **A**: Pie chart representing top 200 genes in ST09 treated PA1 cells that reverted to normal status. **B**: Pie chart representing top op 250 genes in ST09 treated A2780 cells that reverted to normal status (gene list used for A and B are provided in Supplementary table). **C:** Heatmap of the DE genes from ST09 treated A2780 cells depicting their expression pattern in ovarian cancers obtained from GEPIA. **D:** Bar graph depicting reversed expression of these genes upon ST09 treatment in A2780 cells compared to GEPIA ovarian cancer dataset. **E**: Bar graph representing pathways regulated by ST09 treated A2780 (Gene list is provided in the supplementary table). **F:** Bar graph representing pathways regulated by ST09 treated PA1 (Gene list is provided in the supplementary table)


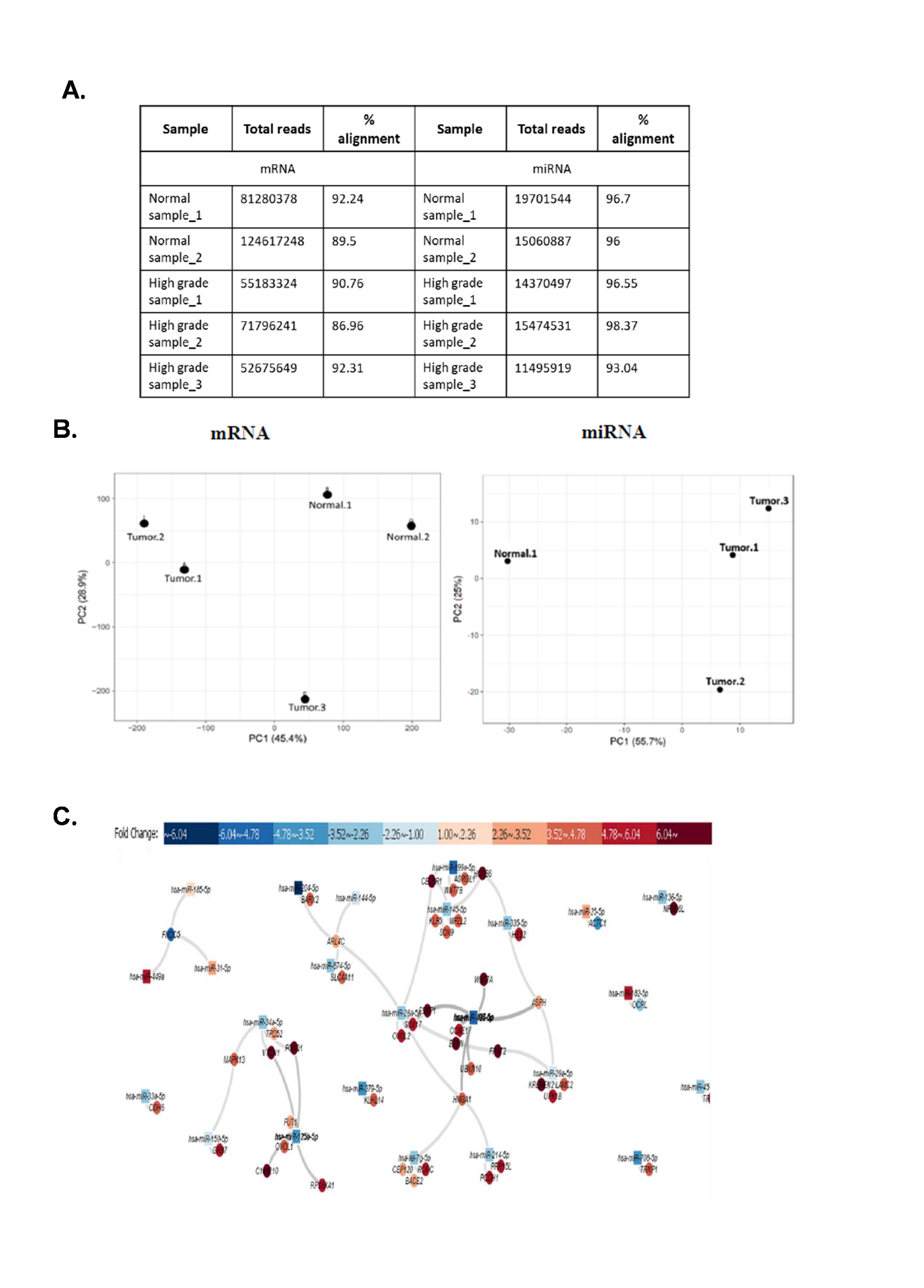


**Supplementary Figure 5: A** Table representing number of reads and alignment percentage of the Indian HGSOC tumor and normal samples. **B** Principle component analysis (PCA) plots for Indian HGSOC tumor and normal samples. **C** Interaction network of anti-correlating mRNA and miRNA pairs in HGSOC tumor samples


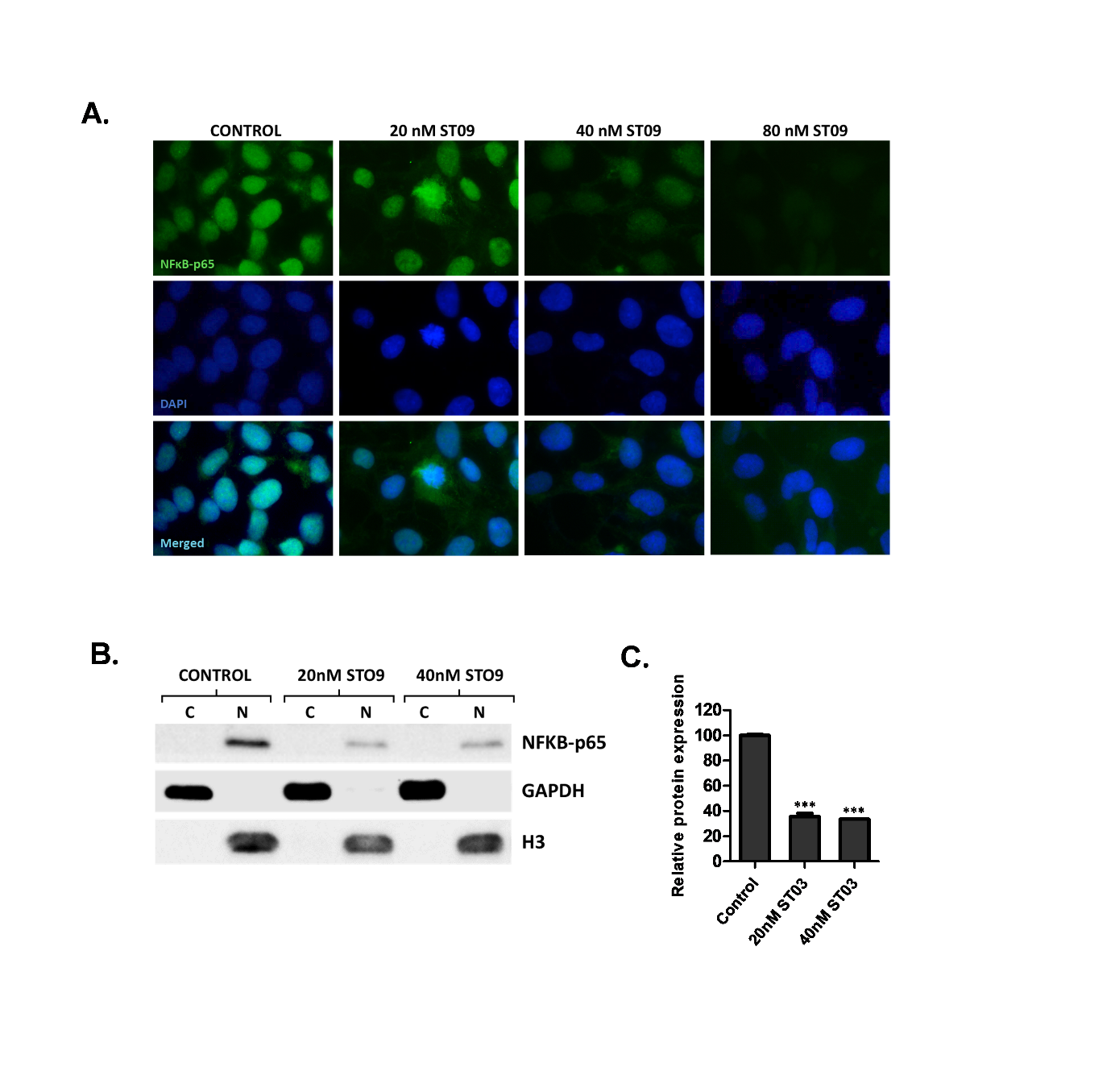


**Supplementary Figure 6: A** Expression of NF-kB upon ST09 treatment in PA1 cells by immunofluorescence analysis. **B** Protein expression of NF-kB in the cytoplasmic versus nuclear fraction in ST09 treatment in PA1 cells. **C** Bar graph quantification of nuclear NF-kB expression with increasing concentrations of ST09 in PA1 cells


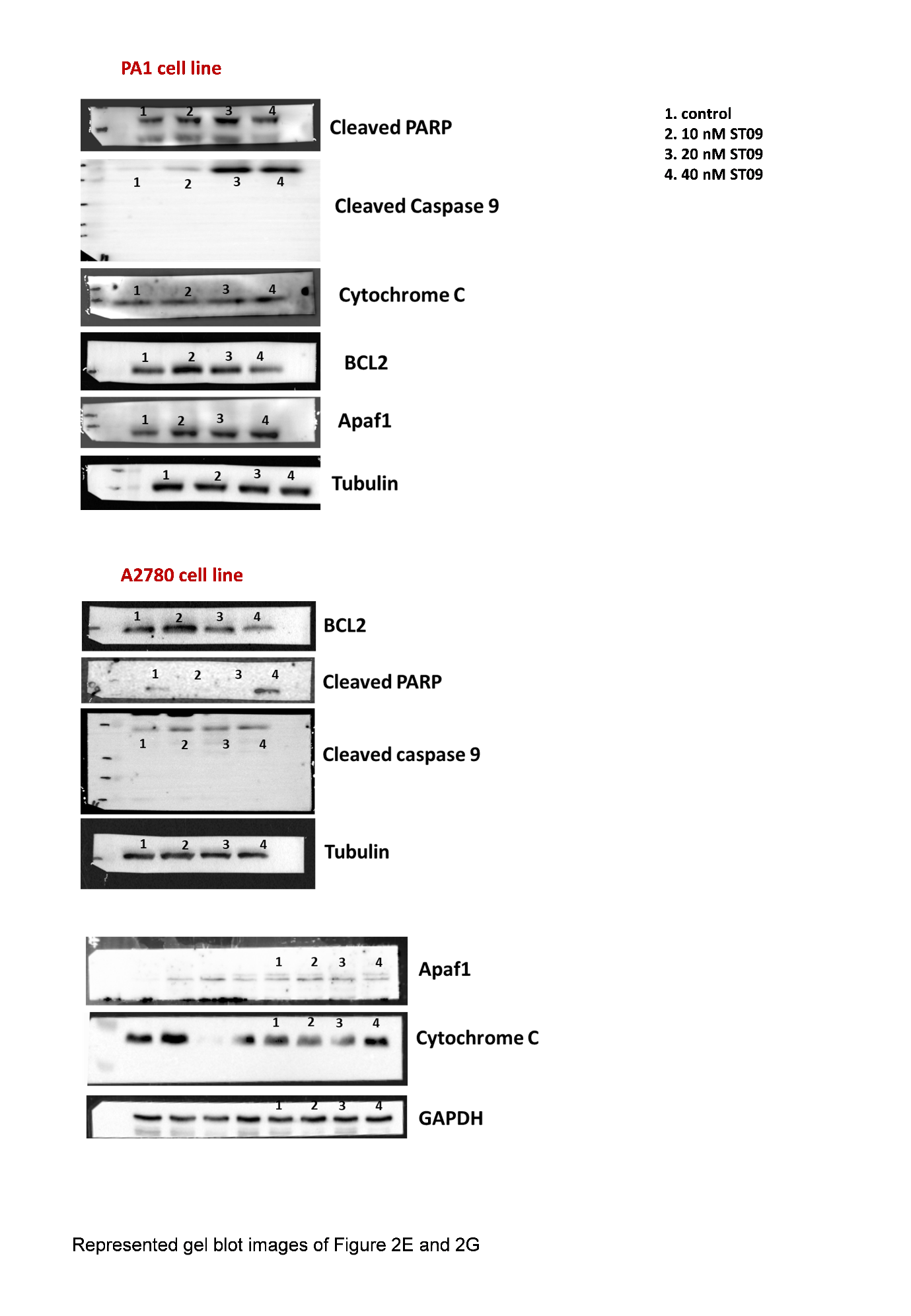


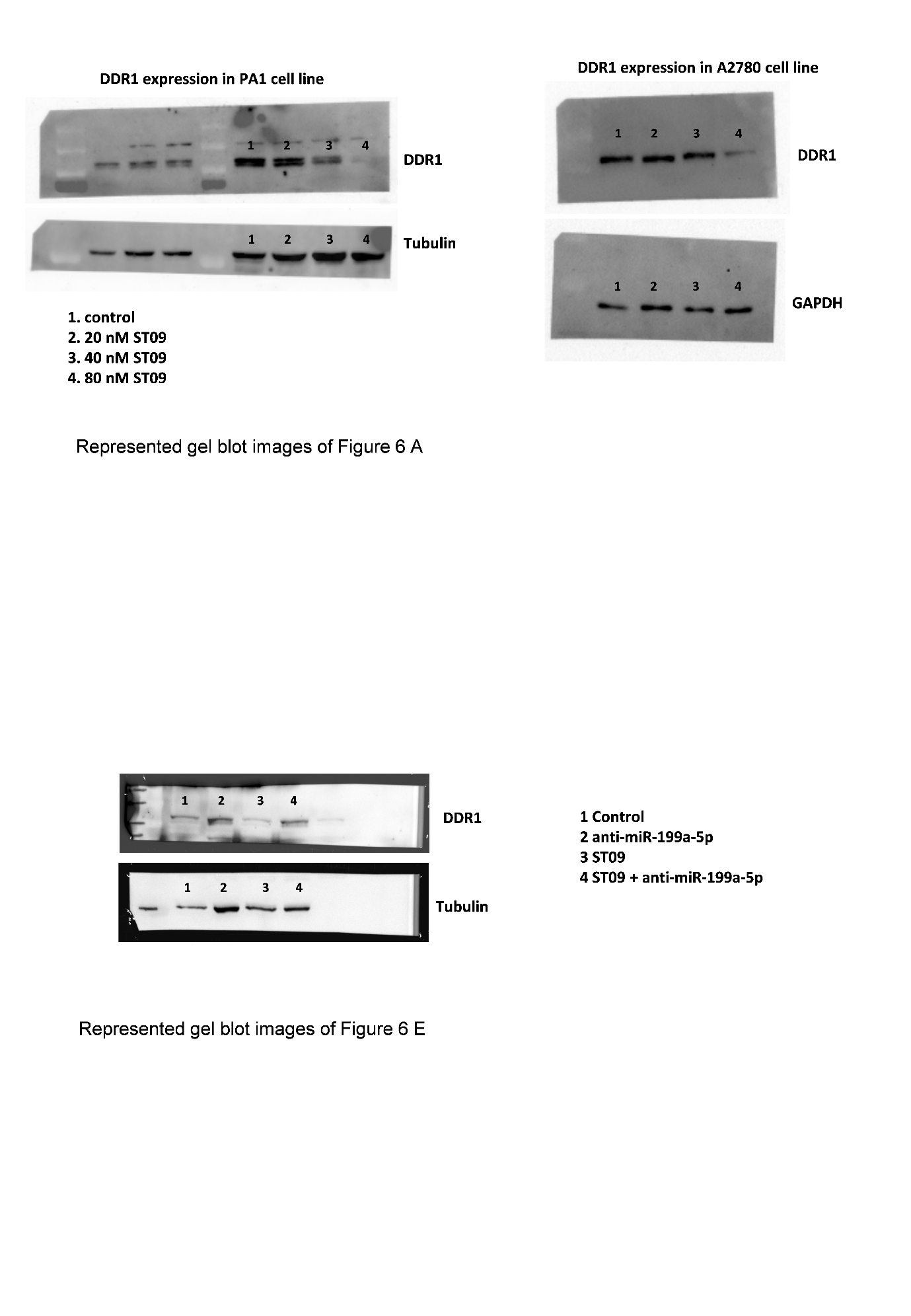


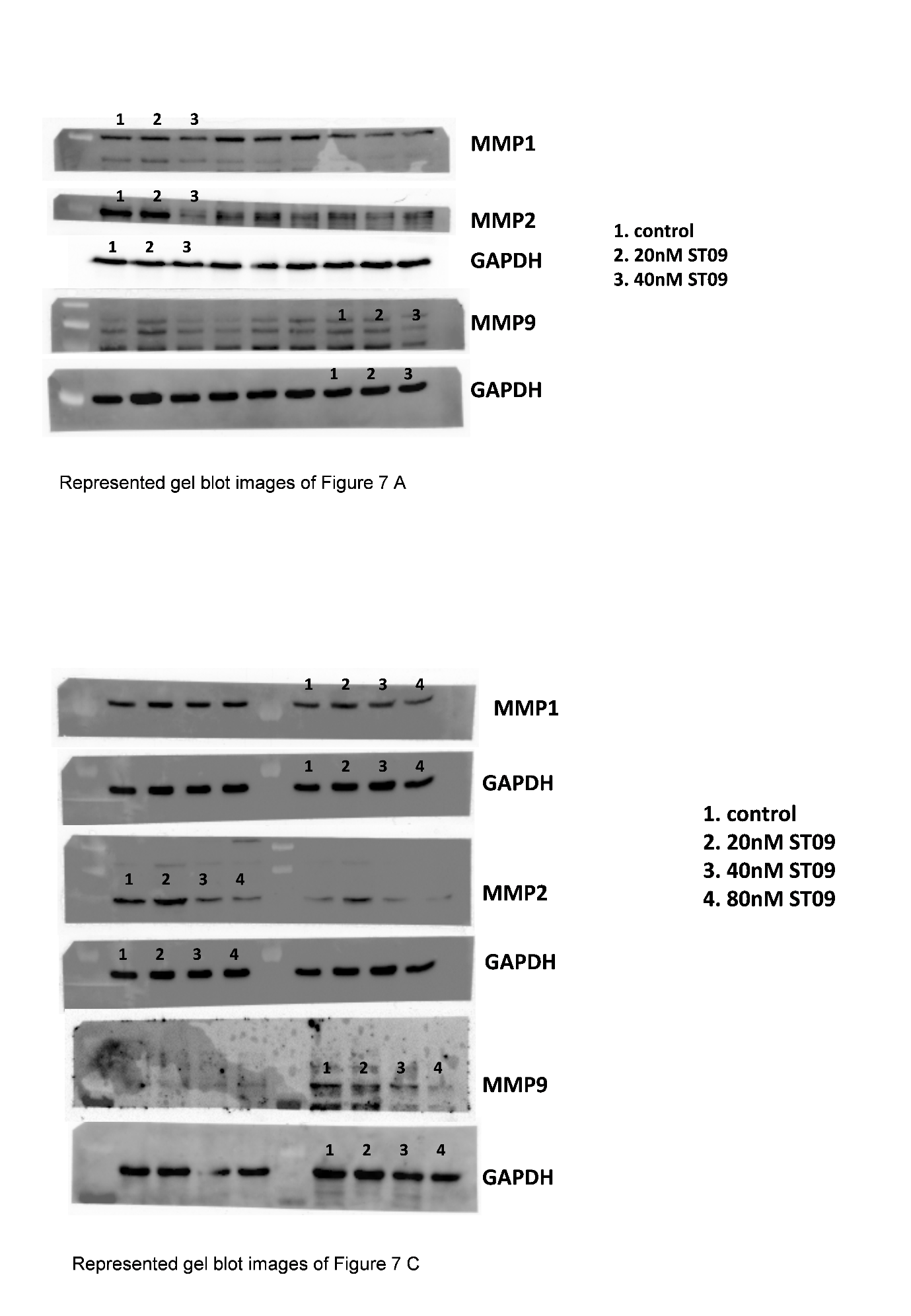


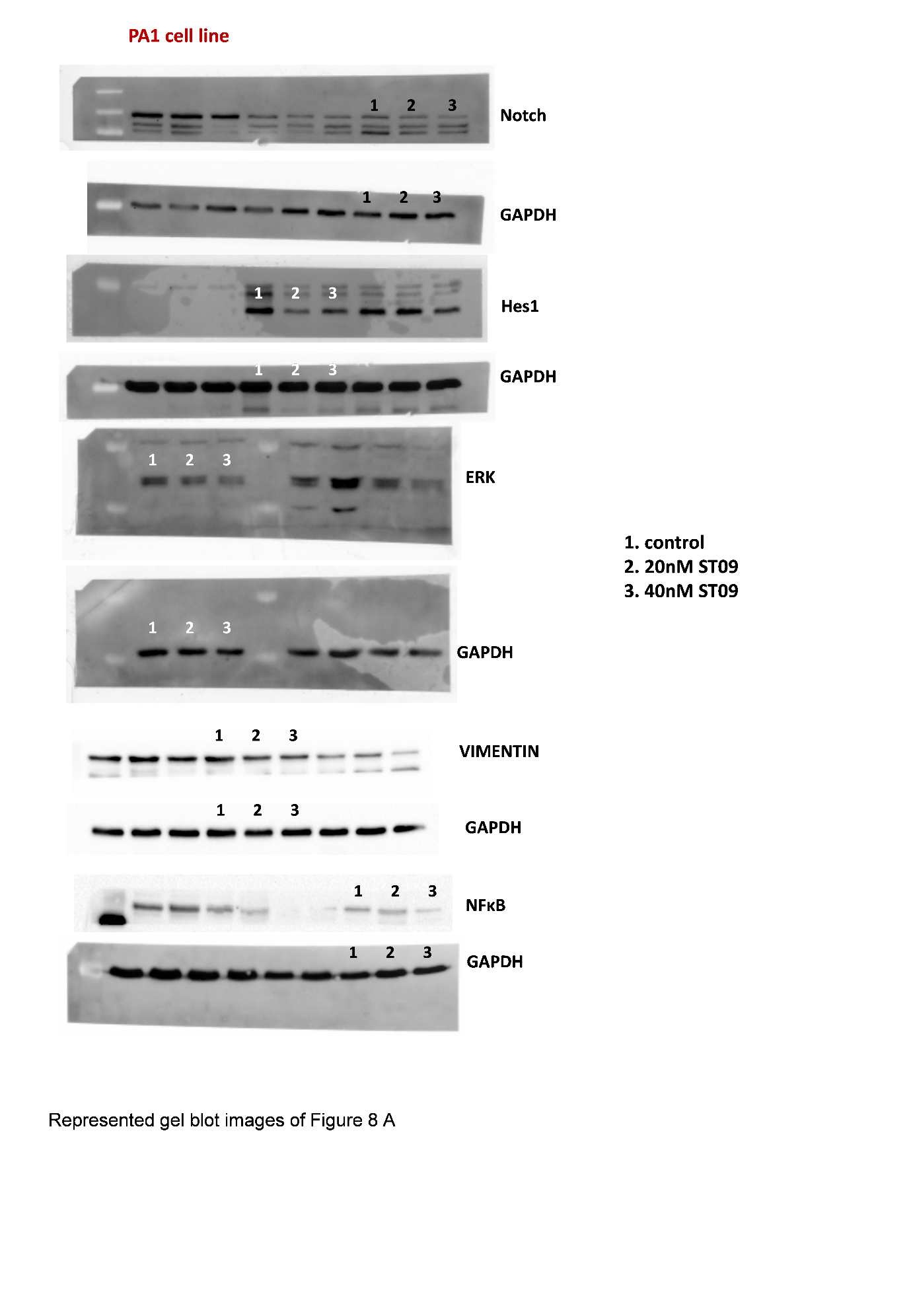


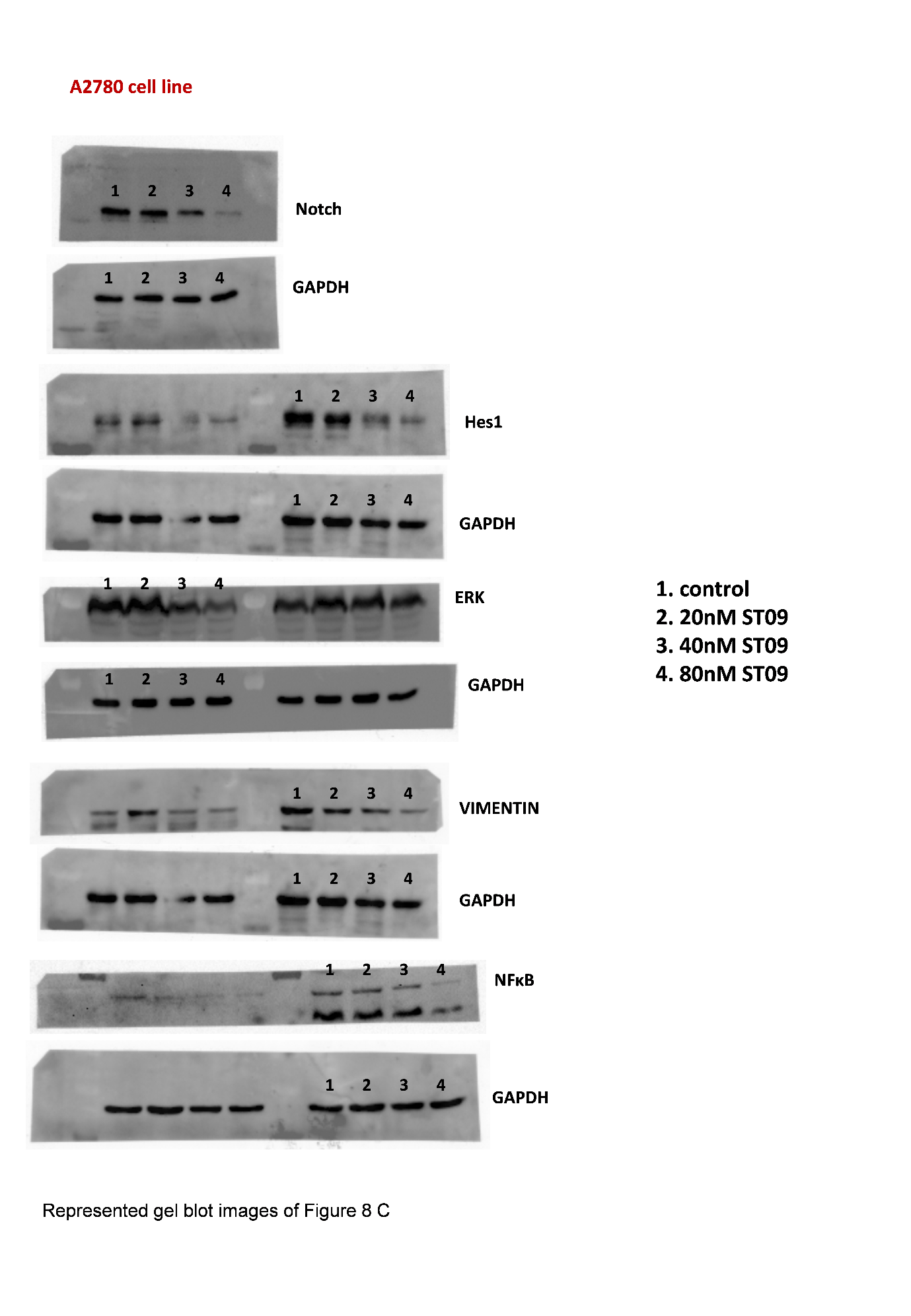


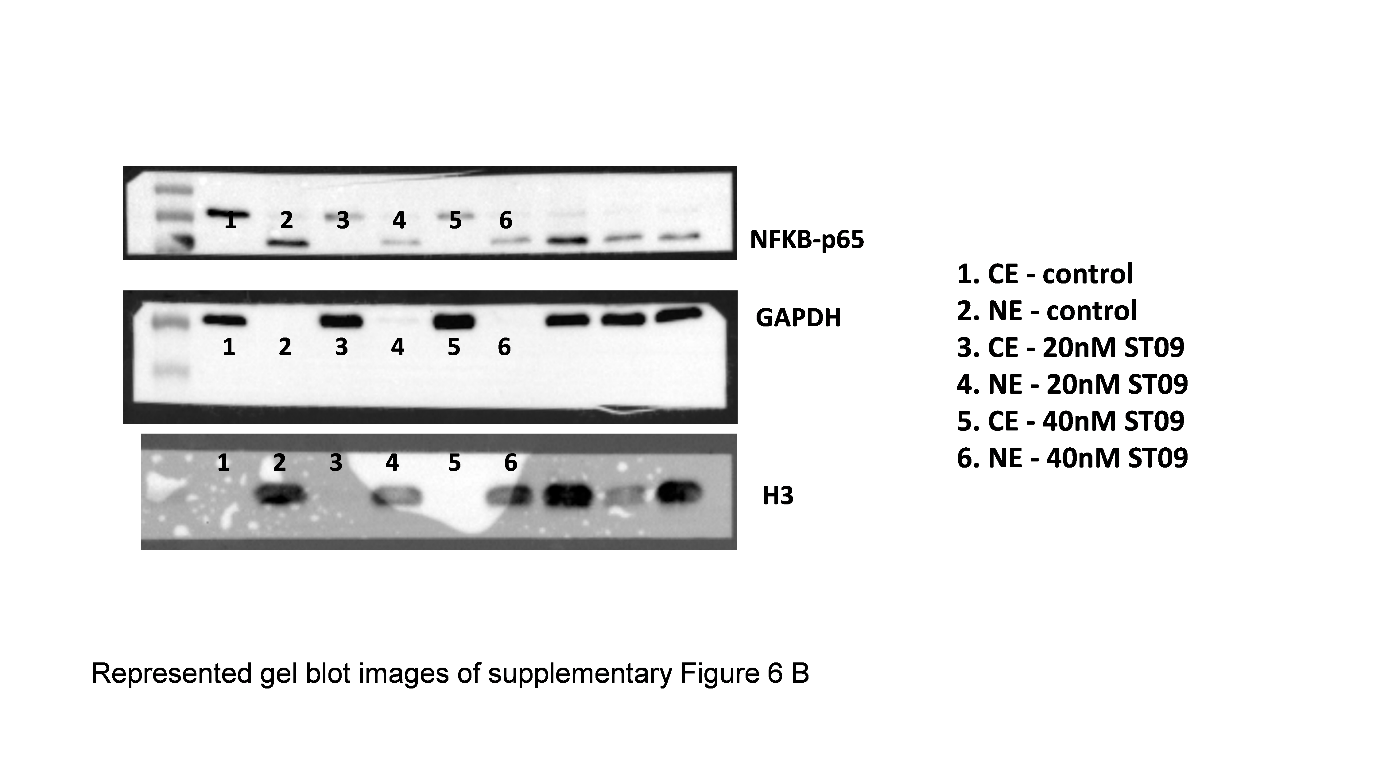

Supplement: Supplementary file 1 — Supplementary Figures. [file 41598_2021_2454_MOESM1_ESM.docx]
